# Efficacy of *Wolbachia-*infected mosquito deployments for the control of dengue

**DOI:** 10.1056/NEJMoa2030243

**Published:** 2021-06-10

**Authors:** Adi Utarini, Citra Indriani, Riris Andono Ahmad, Warsito Tantowijoyo, Eggi Arguni, M. Ridwan Ansari, Endah Supriyati, Dwi Satria Wardana, Yeti Metika, Inggrid Ernesia, Indah Nurhayati, Equatori Prabowo, Bekti Andari, Benjamin R. Green, Lauren Hodgson, Zoe Cutcher, Edwige Rancès, Peter A. Ryan, Scott L. O’Neill, Suzanne M. Dufault, Stephanie K. Tanamas, Nicholas P. Jewell, Katherine L. Anders, Cameron P. Simmons, AWED study group

**Affiliations:** 1 World Mosquito Program Yogyakarta, Centre for Tropical Medicine, Faculty of Medicine, Public Health and Nursing, Universitas Gadjah Mada, Yogyakarta 55281, Indonesia; 2 Department of Health Policy and Management, Faculty of Medicine, Public Health and Nursing, Universitas Gadjah Mada, Yogyakarta 55281, Indonesia; 3 Department of Biostatistics, Epidemiology and Public Health, Faculty of Medicine, Public Health and Nursing, Universitas Gadjah Mada, Yogyakarta 55281, Indonesia; 4 Department of Child Health, Faculty of Medicine, Public Health and Nursing, Universitas Gadjah Mada, Yogyakarta 55281, Indonesia; 5 Department of Biostatistics, School of Public Health, University of California, Berkeley, CA, 94720-1234, USA; 6 London School of Hygiene and Tropical Medicine, Bloomsbury, London, WC1E 7HT, UK; 7 Oxford University Clinical Research Unit, Hospital for Tropical Diseases, 764 Vo Van Kiet, District 5, Ho Chi Minh City, Vietnam; 8 World Mosquito Program, Institute of Vector-borne Disease, Monash University, Clayton, 3800, Australia

## Abstract

**Background:**

*Aedes aegypti* mosquitoes infected with *Wolbachia pipientis* (*w*Mel strain) have reduced potential to transmit dengue viruses.

**Methods:**

We conducted a cluster randomised trial of deployments of *w*Mel-infected *Ae. aegypti* for control of dengue in Yogyakarta City, Indonesia. Twenty-four geographic clusters were randomly allocated to receive *w*Mel deployments as an adjunct to local mosquito control measures; or to continue with local mosquito control measures only. A test-negative design was used to measure efficacy. Study participants were persons 3–45 years old attending primary care clinics with acute undifferentiated fever. Laboratory testing identified virologically-confirmed dengue cases and test-negative controls. The primary endpoint was efficacy of *w*Mel in reducing the incidence of symptomatic, virologically-confirmed dengue, caused by any dengue virus serotype.

**Results:**

Following successful introgression of *w*Mel in intervention clusters, 8144 participants were enrolled; 3721 from *w*Mel-treated clusters and 4423 from untreated clusters. In the ITT analysis virologically-confirmed dengue occurred in 67 of 2905 (2.3%) participants in the *w*Mel-treated and 318 of 3401 (9.4%) in the untreated arm (OR 0.23, 95% CI, 0.15 to 0.35; P=0.004): protective efficacy of 77.1% (95% CI, 65.3 to 84.9). Protective efficacy was similar for the four serotypes. Hospitalisation for virologically-confirmed dengue was less frequent for participants resident in the *w*Mel-treated (13/2905, 2.8%) compared to the untreated arm (102/3401, 6.3%): protective efficacy 86.2% (95% CI, 66.2 to 94.3)

**Conclusions:**

*w*Mel introgression into *Ae. aegypti* populations was efficacious in reducing the incidence of symptomatic dengue, and also led to fewer dengue hospitalisations.

**Trial registration number:**

ClinicalTrials.gov Identifier: NCT03055585 and INA-A7OB6TW

Dengue is a mosquito-borne, acute viral syndrome caused by any of the four serotypes of dengue virus (DENV).^1^ In 2019 the World Health Organisation nominated dengue as one of the top ten global health threats.^2^ Estimates suggest 50–100 million symptomatic cases occur globally each year.^3,4^ Annual (seasonal) and multi-annual epidemic surges in case numbers place considerable pressure on health services in endemic countries.^5^

*Aedes aegypti* mosquitoes are the primary vectors of dengue. Efforts to control *Ae. aegypti* populations using insecticides or environmental management have been the failing standard of care in dengue endemic countries for decades.^6^ Few randomised trials of *Ae. aegypti* control methods have been conducted and none using the gold-standard endpoint of virologically-confirmed dengue.^7^ A trial of community mobilisation to reduce the *Ae. aegypti* population in Nicaragua and Mexico reported modest efficacy (29.5%) against dengue seroconversion in saliva.^8^

*Wolbachia pipientis* are common, maternally-inherited, obligate intracellular bacteria that infect many species of insects but do not naturally occur in *Ae. aegypti* mosquitoes.^9^ Stable transinfection of *A*. *aegypti* with some strains of *Wolbachia* confers on the mosquito resistance to disseminated infection by DENV and other arboviruses.^10–13^ Thus, the introgression of “virus blocking” strains of *Wolbachia* into field-populations of *Ae. aegypti* is an emerging dengue control method.^14–17^ The approach works by delivering regular pulses of *Wolbachia*-infected mosquitoes into the wild mosquito population over a period of several months. Helpfully, *Wolbachia* self-propels its own population introgression by manipulating reproductive outcomes between wild-type and *Wolbachia*-infected mosquitoes – the only viable mating outcomes are those where the progeny are *Wolbachia*-infected.^13^

Here we report results of a city-wide cluster randomised trial to measure the efficacy of *Wolbachia* (*w*Mel strain)-infected mosquito deployments in reducing the incidence of virologically-confirmed dengue in Yogyakarta City, Indonesia. The trial builds on earlier entomological and epidemiological pilot studies in this setting.^14,18,19^

## Methods

### Trial design and oversight

The “Applying Wolbachia to Eliminate Dengue” (AWED) trial was financially supported by the Tahija Foundation and the trial sponsor was the Universitas Gadjah Mada, Indonesia. The protocol was published^20,21^ and is provided in the Supplementary Appendix at nejm.org.

Community support for randomised *w*Mel releases was obtained from leaders of 37 urban villages following a community engagement and mass communications campaign. For enrolment into the clinical cohort in primary health care facilities, written informed consent was obtained from all participants or their guardian where the participant was a minor. In addition, participants aged between 13 and 17 years gave written informed assent to participate. The trial was conducted in accordance with the principles of the Good Clinical Practice guidelines of the International Conference on Harmonisation and was approved by the Universitas Gadjah Mada and the Monash University Human Research Ethics Committees. The trial data was analysed by the independent trial statisticians (NPJ and SMD). The funders had no role in the analysis of the data, in the preparation or approval of the manuscript, or in the decision to submit the manuscript for publication.

### Randomisation

**Figure 1:**
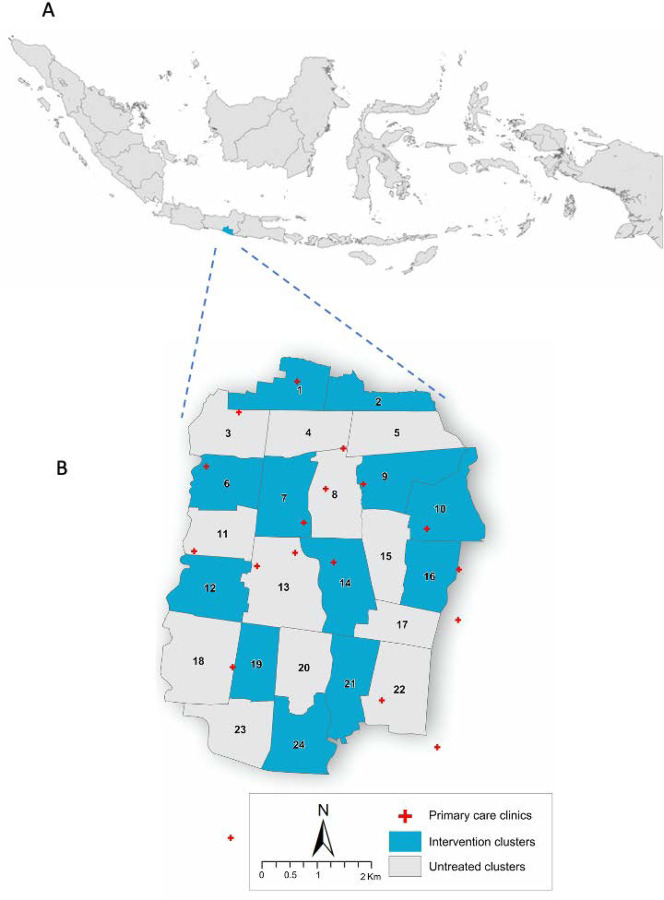
Map of study location. In panel A, the map of Indonesia is shown with the Special Region of Yogyakarta shaded blue. In panel B, the map of Yogyakarta City (plus a small region of neighbouring Bantul District) is shown with *w*Mel intervention clusters (shaded blue) and untreated clusters (shaded grey) indicated. The locations of primary care clinics (red crosses) where enrolment occurred are also shown.

The baseline characteristics of the study site are described in Table S1. Briefly, the study site was a continuous urban area of 26 km^2^ and with a population of approximately 311,700. The study site was subdivided into twenty-four contiguous clusters, each approximately 1km^2^ in size and where possible having borders that would slow the dispersal of mosquitoes between clusters. Of the 24 clusters, 12 were randomly allocated to receive open label *Wolbachia* deployments and 12 left untreated (Figure 1 and Figure S1). In treated clusters most community members will have been unaware of treatment assignment because mosquito release containers were discreetly placed in a minority of residential properties for a time-limited period in 2017 (Table S2). No placebo was used in the untreated arm. Constrained randomisation was used to prevent a chance imbalance in the baseline characteristics or spatial distribution of treated and untreated clusters (described in Supplementary Appendix).

### Wolbachia deployment and entomological monitoring

*w*Mel-infected *Ae. aegypti* were sourced from an outcrossed colony described previously.^14^ As expected, these *w*Mel-infected mosquitoes had reduced transmission potential for DENV compared to wild-type *Ae. aegypti*, as described in the Supplementary Appendix and Figures S2 and S3. Mosquitoes were released as eggs into intervention clusters between March and December 2017. Each cluster received between 9–14 rounds of releases (see Table S2 for details). Mosquito releases, and the monitoring of *w*Mel frequencies via a network of 348 BG-Sentinel adult mosquito traps (BioGents), are described in the Supplementary Appendix.

### Participant enrolment

Participant enrolment to measure the efficacy endpoint was performed at a network of 18 government-run primary care clinics in Yogyakarta City and adjacent Bantul District.

Patients presenting to the clinics were eligible for the study if they met the inclusion criteria, a) fever (either self-reported or objectively measured, defined as forehead or axillary temperature >37.5°C) with a date of onset between 1–4 days prior to the day of presentation, b) aged between 3–45 years old and c) resided in the study area every night for the 10 days preceding illness onset. Participants were not eligible if they a) had localising features suggestive of a specific diagnosis other than an arboviral infection, e.g. severe diarrhea, otitis, or pneumonia, or b) were enrolled in the study within the previous 4 weeks.

### Procedures

Enrolled participants provided demographic information, geolocated residential address and a detailed travel history (durations and geolocations) for the past 10 days. A 3 ml venous blood sample was collected for arbovirus diagnostic tests. No other diagnostic investigations were performed. Participants were followed up 14–21 days later to determine whether they a) were alive, and b) had been hospitalised since their enrolment in the study. No information on the clinical severity of virologically-confirmed dengue (VCD) cases was acquired No further information on disease severity or clinical diagnoses was acquired.

### Diagnostic investigations and classifications

Study participants were classified as VCD cases if their enrolment plasma sample was DENV test-positive in a multiplex (DENV, chikungunya and Zika virus) reverse-transcriptase polymerase chain reaction (RT-PCR) and/or in an enzyme-linked immunosorbent assay (ELISA) for dengue NS1 (BioRad Platelia). Study participants were classified as test-negative controls if their enrolment plasma sample was test-negative by RT-PCR for DENV, chikungunya and Zika viruses, and also test-negative for DENV NS1 and negative in dengue IgM and IgG capture ELISAs. The diagnostic algorithm is shown in Figure S4. Note that the DENV serotype was determined via a 2^nd^ independent RT-PCR test (Simplexa) by an independent laboratory at the Eijkman Institute, Jakarta. Details of the diagnostic methods are provided in the Supplementary Appendix.

### Primary and secondary endpoints

The primary endpoint was the efficacy of community-based deployments of *w*Mel-infected *Ae. aegypti* mosquitoes in reducing the incidence of symptomatic, virologically-confirmed dengue cases of any severity in Yogyakarta residents aged 3–45 years in release (intervention) areas, relative to non-release (untreated) areas. Secondary endpoints reported here include the efficacy against each of the four DENV serotypes.

### Sample Size

Reflective of the novel design, the sample size requirements to demonstrate a 50% reduction in dengue incidence, which was considered the minimum effect size for public health value, evolved over time. The full sample size narrative is provided in the Supplementary Appendix. Briefly, 400 VCD cases and four times as many controls was determined to be sufficient to detect a 50% reduction in VCD case incidence with 80% power. The emergence of SARS-CoV-2 in Yogyakarta in March 2020 prevented the continued enrolment of participants in clinics, with enrolment stopping on 18^th^ March 2020. On 5^th^ May 2020, the trial steering committee endorsed the recommendation from the trial investigators to terminate the trial having recruited 385 VCD cases.

### Statistical Analysis

The statistical analysis plan was published^22^ and is available in the Supplementary Appendix. The dataset for analysis included all enrolled VCD cases and all test-negative controls, excluding participants enrolled prior to *Wolbachia* establishment throughout intervention clusters (defined as one month after completion of releases in the last cluster) and excluding test-negative controls enrolled in a calendar month with no enrolled dengue cases. The primary intention-to-treat (ITT) analysis considered *Wolbachia* exposure as a binary classification based on residence in a cluster allocated to *Wolbachia* deployment or not. Residence was defined as the primary place of residence during the 10 days prior to illness onset. The intervention effect was estimated from an aggregate odds ratio (OR) comparing the exposure odds (residence in a *Wolbachia*-treated cluster) among VCD cases versus test-negative controls, using the constrained permutation distribution as the foundation for inference. The null hypothesis was that the odds of residence in a *Wolbachia*-treated cluster was the same among VCD cases as test-negative controls. Efficacy of the intervention was calculated as 100*(1-aggregate OR). A predefined exploratory analysis evaluated the efficacy of the intervention in preventing hospitalised virologically-confirmed dengue cases.

An additional pre-defined cluster-level ITT analysis was performed by calculating the VCD case proportion in each cluster. The difference in the average proportion of VCD cases between the intervention clusters and untreated clusters was used to test the null hypothesis of no intervention effect (a t-test statistic) and to derive an estimate of the cluster-specific relative risk, with inference based on the constrained permutation distribution.^23,24^

The same intention-to-treat analyses described above were applied for the secondary endpoint of serotype-specific efficacy, with case populations restricted to each of the DENV serotypes in turn, and with the same test-negative control population as for the primary analysis.

Per protocol analyses considered exposure contamination by assigning a *Wolbachia* exposure index to each participant based on the *w*Mel frequency in their cluster of residence only, or by combining this frequency with the participant’s recent travel history. A generalized linear model was fitted, with balanced bootstrap resampling based on cluster membership, to estimate the relative risk of VCD and associated confidence interval in each quintile of *Wolbachia* exposure, relative to baseline. Detailed methods are provided in the Supplementary Appendix.

## Results

### Establishment of wMel in Ae. aegypti populations

**Figure 2:**
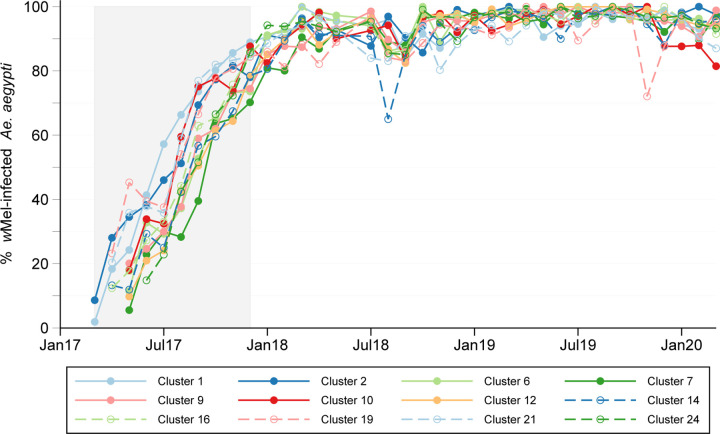

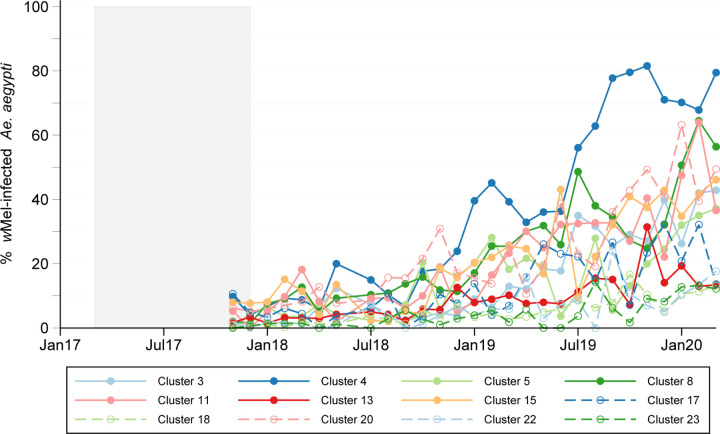
*w*Mel introgression into local *Aedes aegypti* mosquito populations. Lines show the percentage of *Ae. aegypti* collected from intervention clusters (A) and untreated clusters (B) that were *w*Mel infected, each month from the start of deployments (March 2017) to the end of participant enrolment (March 2020). The shaded area indicates the period from the first release in the first cluster (March 2017) to the last release in the last cluster (December 2017). There were between 9 and 14 fortnightly release rounds per cluster.

This trial was performed in Yogyakarta, Indonesia (Figure 1). *w*Mel was durably established in the *Ae. aegypti* populations in each of the 12 intervention clusters (Figure 2). The monthly median (interquartile range) cluster level *w*Mel prevalence was 95.8% (91.5–97.8%) during the 27 months of clinical surveillance.

### Study participants

**Figure 3:**
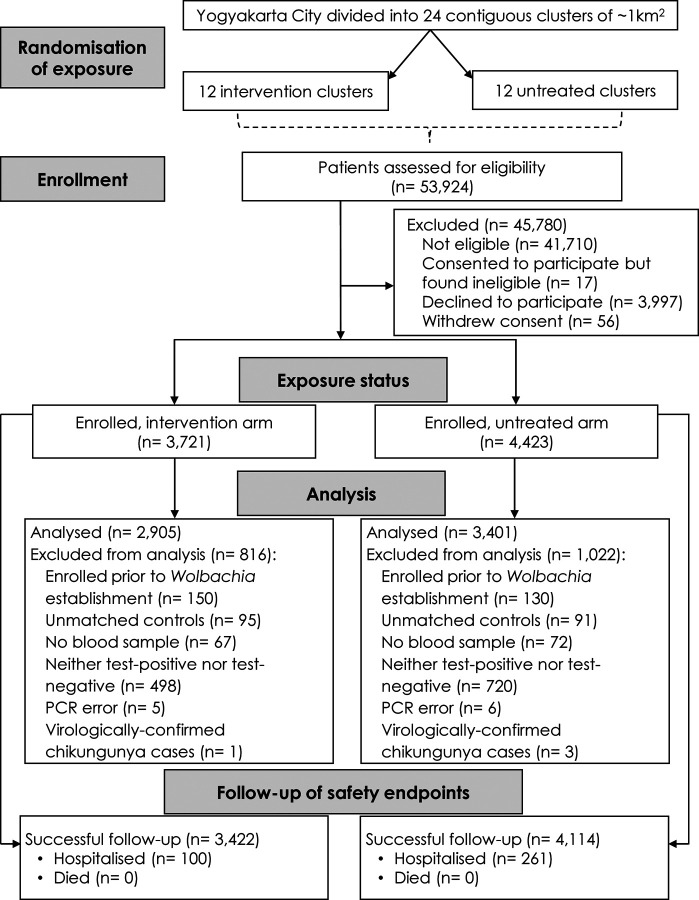
Cluster randomisation, participant enrolment, inclusion in analysis dataset, and follow-up of safety endpoints. The commonest reasons for exclusion from the analysis dataset were enrolment before the predefined time point of *Wolbachia* establishment (8^th^ January 2018), enrolment in a calendar month without any VCD cases (September 2018) or having positive or equivocal dengue IgM or IgG serology at enrolment that precluded classification as a test-negative control.

53,924 patients were screened for study eligibility at 18 primary care clinics between January 8^th^ 2018 and March 18^th^ 2020 and 8144 persons were enrolled. Of these, 6306 participants met the requirements for the primary analysis dataset; 2905 participants were resident in the *w*Mel intervention arm and 3401 in the untreated arm (Figure 3). Four virologically-confirmed chikungunya cases (1 in the *w*Mel-treated arm and 3 in the untreated arm) were excluded from the primary analysis dataset. No Zika cases were detected. The median age (interquartile range) of participants was 11.6 years (6.7, 20.9) and 48.8% of participants were female (Table S3). 295 (4.7%) of the 6306 participants in the analysis dataset were hospitalised in the time between their enrolment and follow-up 14–21 days later. Hospitalisation was significantly less frequent for participants resident in the *w*Mel-treated arm (81/2905, 2.8%) compared to the untreated arm (214/3401, 6.3%) (OR 0.43, 95% confidence interval [CI], 0.32 to 0.58; P=0.004) (Table S4). This lower probability of hospitalisation was evident across the clinic network (Figure S5). 385 (6.1%) of 6306 participants in the analysis dataset were VCD cases and 5921 (93.8%) were test-negative controls. VCD cases and test-negative controls were well-matched by age and gender (Table S3).

### Intention to treat analyses

**Figure 4:**
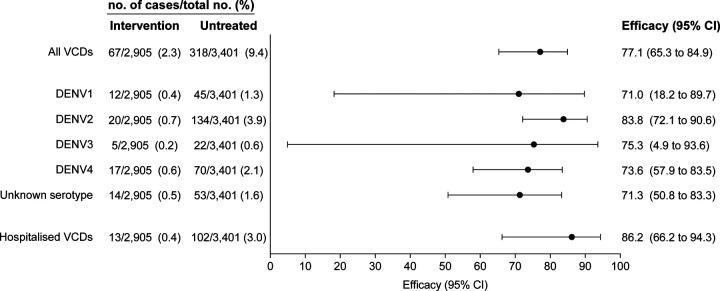
Intention-to-treat efficacy. Shown is the protective efficacy (expressed as 100×(1−OR)) of *w*Mel-infected *Aedes aegypti* deployments against virologically-confirmed dengue of any serotype (All VCDs), by infecting DENV serotype, and against hospitalised VCD. VCDs with ‘Unknown serotype’ were test-negative by DENV RT-PCR and test-positive for DENV NS1 antigen. Seven participants had two DENV serotypes detected during the same febrile episode: four with serotypes 1 and 2, two with serotypes 1 and 4, and one with serotypes 2 and 4.

The incidence of VCD cases was significantly lower in the *w*Mel-treated arm (67 VCDs amongst 2905 participants (2.3%)) than in the untreated arm (318/3401 (9.4%)) (OR 0.23, 95% CI, 0.15 to 0.35; P=0.004). This represented a protective efficacy of 77.1% (95% CI, 65.3 to 84.9) (Figure 4). The intervention effect was evident by 12 months after *w*Mel-establishment (Figure S6). Protective efficacy was similar across serotypes, being highest for DENV-2 (83.8%; 95% CI, 72.1 to 90.6) and lowest for DENV-1 (71.0%; 95% CI, 18.2 to 89.7) (Figure 4). For all four serotypes the lower bound of the 95% CI for protective efficacy was greater than 0. There were 13 hospitalisations for VCD amongst 2905 participants (0.4%) from the *w*Mel treated arm compared to 102 hospitalisations for VCD amongst 3401 participants (3%) from the untreated arm, for a protective efficacy of 86.2% (95% CI, 66.2 to 94.3) (Figure 4 and Table S5).

**Figure 5:**
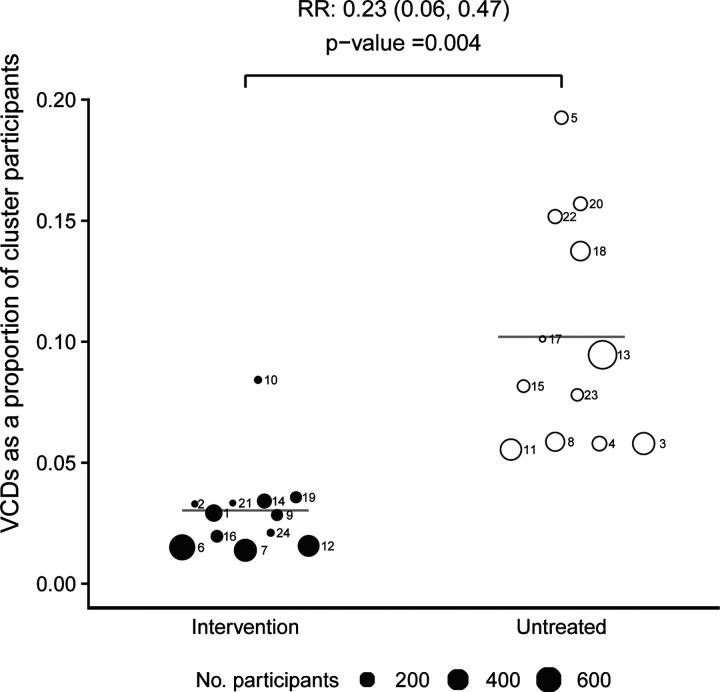
Cluster-level proportions of virologically-confirmed dengue cases. VCD cases as a proportion of all participants in *Wolbachia*-treated (closed circles) and untreated (open circles) clusters. Circle size is proportionate to the total number of participants in the cluster. Circles are labelled with their respective cluster number. Horizontal bars show the mean VCD proportion in intervention and untreated clusters; the relative risk and P-value are derived from a comparison of these mean proportions (see Methods).

An additional prespecified ITT analysis compared VCD cases as a proportion of total participants in each cluster, between study arms. In all but one of the *w*Mel-treated clusters the VCD proportion was lower than untreated clusters, yielding a relative risk of 0.23 (95% CI, 0.06 to 0.47; P=0.004) (Figure 5). Figure S7 shows the VCD proportion and *w*Mel prevalence over time in individual clusters. When stratified by serotype, the relative risk of VCD caused by the two most prevalent serotypes, DENV-2 and DENV-4, was significantly lower in the *w*Mel-treated arm (Figure S8).

### Per protocol analyses

Per protocol analyses assigned a *Wolbachia* exposure index to each participant based on the *w*Mel frequency in their cluster of residence only, or by accounting also for *w*Mel frequencies and time spent in other locations. Protective efficacy against VCD increased with incremental increases in participants’ *Wolbachia* exposure index when cluster of residence and recent travel history were considered (Figure S9A). When only the *w*Mel frequency in the cluster of residence was considered a threshold effect was observed in that only cluster-level *w*Mel frequencies >80% were protective (Figure S9B).

## Discussion

Establishment of *w*Mel in *Ae. aegypti* mosquitoes in Yogyakarta reduced the incidence of symptomatic VCD amongst 3–45 year olds by 77%. Reassuringly, protective efficacy was observed against all four DENV serotypes, with greatest confidence for DENV-2 and -4 as these were the most prevalent serotypes. Efficacy against VCD requiring hospitalisation, a pragmatic proxy of clinical severity, was 86%. Eleven of the twelve *w*Mel treated clusters had a lower proportion of VCD cases than untreated clusters, demonstrating consistent biological replication of the intervention effect.

The conceptual underpinnings of the test negative design used in this trial, and the statistical framework for population inference, have been described.^23^ Acute undifferentiated fever of one to four days duration was set as the clinical basis for participant eligibility to avoid selection bias at the point of recruitment and to enable virological detection of dengue cases. Trial operational procedures, particularly blinding of research staff, aimed to prevent bias in follow-up, laboratory testing and outcome classification. The intervention (mosquito releases) was delivered openly, and not placebo controlled, for several months in each cluster during 2017. There was no evidence this changed the health care seeking behaviour of community members in subsequent years because similar numbers of participants meeting the eligibility criteria were enrolled from each arm of the trial.

*w*Mel-infected mosquito populations were not static and spatially heterogenous *w*Mel contamination was measured at the edges of untreated clusters in year two of the trial. Nonetheless the efficacy estimates from per protocol analyses, which accounted for individual participants’ recent exposure to *w*Mel via changes in cluster-level *w*Mel frequencies and/or human movement, did not exceed that measured in the ITT analysis. We plan more nuanced exploratory analyses, outside the scope of the current protocol, to explore the fine spatial and temporal connections between *w*Mel prevalence and risk of VCD.

The efficacy results reported are consistent with a body of laboratory and field observations. Predictions from an ensemble of mathematical models have suggested that the reduced infectiousness observed in *w*Mel-infected *Ae. aegypti* could be sufficient to reduce R0 (the basic reproductive number) to below one in many dengue endemic settings, which could result in local elimination of disease.^3,25,26^ Previous non-randomised field studies in Australia^16,17^ and Indonesia^14^ provided evidence of large epidemiological impacts after *w*Mel was introgressed. A quasi-experimental study of *Wolbachia* deployments in seven urban villages on the northwestern border of Yogyakarta, compared to three untreated control villages on the southeastern border of the city, reported a 76% reduction in the incidence of hospitalised dengue hemorrhagic fever during 30 months post-deployment.^14^ Together with the trial reported here, this body of work suggests that when *w*Mel is established at high prevalence in local *Ae. aegypti* populations then reductions in dengue incidence follow. Another *Wolbachia* strain, *w*AlbB, also has pathogen-blocking properties and can be introgressed into *Ae. aegypti* field populations.^15^ This suggests the possibility of a portfolio of *Wolbachia* strains, each with different strengths and weaknesses, for application as public health interventions in dengue endemic areas.

Stable *w*Mel transinfection imparts a viral resistant state in *Ae. aegypti* mosquitoes that attenuates superinfection by several medically-important *Flaviviruses* and *Alphaviruses*. Multiple mechanisms have been proposed to explain this phenotype, including *Wolbachia*-induced triggering of innate immune effectors^27,28^ and changes in intracellular cholesterol transport.^29^ DENV could plausibly evolve resistance to *w*Mel however the requirement for alternating infection of human and mosquito hosts, together with what appears to be a complex mode of action, could be a constraint on the adaptive emergence of resistant virus populations. Future research should survey arbovirus populations for signals of *Wolbachia*-associated selective pressure.

The *w*Mel introgression approach represents a novel product class for the control of dengue.^30^ An attractive aspect of this strategy is that it maintains itself in the mosquito population and does not need re-application.^31^ Future trials should explore the multivalency of the intervention, since laboratory studies^12,32–35^ suggest *w*Mel should also attenuate transmission of Zika, chikungunya, Yellow Fever and Mayaro viruses by *Ae. aegypti*.

## Supplementary Material

1
