# Supplementary material for: Efficacy of Wolbachia-infected mosquito deployments for the control of dengue
Source: N Engl J Med. Author manuscript; Available in PMC 2021 Jun 10. (PMC8103655; doi:10.1056/NEJMoa2030243)
Supplement: 1 [file 2030243-Supplement.pdf]

Efficacy of *Wolbachia*-infected mosquito deployments for the control of dengue

Supplementary appendix

Contents

AWED Study committees 2

AWED Study Group 2

Supplementary Methods 4

Constrained randomisation procedure 4

*w*Mel-infected *Aedes aegypti* mosquito production 8

Vector competence experiments using *w*Mel-infected *Ae. aegypti* from Yogyakarta 8

*w*Mel-infected *Aedes aegypti* deployments 9

Entomological monitoring 9

Arbovirus diagnostic investigations 10

Sample size calculations 11

Calculation of *Wolbachia* Exposure Index for per-protocol analysis 14

Per-protocol analysis statistical methods and results 15

Supplementary Tables 16

Table S1: Baseline characteristics of clusters (sociodemographic, historical disease incidence, area and mosquito trapping) 16

Table S2: Summary of *Wolbachia* deployments in 12 intervention clusters 18

Table S3: Baseline characteristics of trial participants 19

Table S4: Hospitalisation outcome by treatment arm for all participants in the analysis dataset 20

Table S5: Hospitalisation outcome by treatment arm for VCD cases 20

Supplementary Figures 21

Figure S1: Map of study location with satellite overlay. 21

Figure S2. Susceptibility of wild-type [WT] and wMel-infected Ae. aegypti to DENV infection. 22

Figure S3: Viral load in mosquito abdomen tissue per mosquito by strain (*w*Mel vs wild-type [WT]) and serotype. 23

Figure S4: Diagnostic algorithm and classification of participants 24

Figure S5: Participant enrolment and hospitalisations by primary care clinic and treatment arm. 25

Figure S6: Intervention efficacy over time. 26

Figure S7: Cluster-level proportions of virologically-confirmed dengue cases and prevalence of *w*Mel in local *Aedes aegypti* mosquito populations over time. **Error! Bookmark not defined.**

Figure S8: Cluster-level proportions of serotype-specific virologically-confirmed dengue cases. 30

Figure S9: Efficacy of the *Wolbachia* intervention against virologically-confirmed dengue according to *Wolbachia* exposure index (per-protocol analysis). 31

AWED Study committees

Trial Steering Committee members
Siswanto; National Institute of Health Research and Development, Ministry of Health, Indonesia
Tedjo Sasmono; Eijkman Institute, Jakarta, Indonesia
Pembayun Setyaning Astuti, Yogyakarta Provincial Health Office, Indonesia

Independent Data Monitoring Committee members
Immo Kleinschmidt, London School of Hygiene and Tropical Medicine, UK
Sri Rezeki Syaraswati Hadinegoro, Universitas Indonesia, Indonesia
Ooi Eng Eong, Duke-NUS Medical School, Singapore
In Kyuu Yoon, Coalition for Epidemic Preparedness Innovations (CEPI), USA
Anna P Durbin, Johns Hopkins Bloomberg School of Public Health, USA.

Trial statisticians
Nicholas Jewell, University of California, Berkeley, USA & London School of Hygiene and Tropical Medicine, UK
Suzanne Dufault, University of California, Berkeley, USA

AWED Study Group

Stakeholder and Community Engagement
Equatori Prabowo, Ranggoaini Jahja, Achmad A. Tamrin, Sylva Haryosaputro, Muhammad Ali Mahrus, Dedik Helmy Yusdiana, Gito Haryanto, Susi Dewi Nova Riyanti, Didit S. Munandar, Kiswi Radat, Maria Bernadette Rindiyastami, Uswatun Khasanah, Rini Kurniati, Ismail Hidayat, Florentinus Dhanu Nugroho, Iyxda Dwi Hartanto, Nur Solikhah, Bekti Andari.

Entomology

Indah Nurhayati, Iva Fitriana,Budi Arianto,Nida Budiwati Pramuko, Sigit Setyawan, Anastasia Maya Indah Lestari, Wasini, Bambang Hardiko Nugroho, Heru Sulistyo, Kiswanto, Murniwati Handayani, Rizki Sholeh, Tri Mumpuni, Anis Ariestanto, Yusqi Taufiqur Rohman, Tilka Hujjatuna, Merry Putri Wijayanti, Resita Octiani, Sri Yuliani Dewi, Desy Liana, Indira Diah Utami, Anisa Parazulfa, Chandra Paditaningrum, Nur Fauziah, Zahtamal, Endang Pramesthiningsih Nabhela Ayu Purwaningrum, Hery Agus Susilo, Ita Ayundasari, Puji Rahayu, Sularto, Trismiyatun, Wahyu Pranoto, Noor Cholivah, Reza Yustian, Defriana Lutfi Chisnaifah, Rifqi Zahro Janatunaim

Clinical research
Uswatun khasanah, Ragil Eva Agustin, Muhammad Tamaji, Nurina Jihan Yulianti, Nur Khasanah,Munasdi Victorius, Ktut Rentyasti Palupi, Ardhina Ramania, Maryati, Sulis Mukaryanah Widarti, Rini Dwi Utami, Siti Rochmatun, Oktalia Nurfita Sari, Mugiyanti Ikawati, Shofia Eva Sagita, Imam Fungani, Pramidya Ujiana, Siti Nuryanti, Laila Latifah, Eka Rahayuningsih, Roseta Irawati, Fatika Ikhtariyani, Arief Rachman Hakim, Wahyuningsih, Tri Kunarsih, Anggun Hatto Hanti, Dwy Desy Rahmawati, Fitri Ramadhan, Fradina Kiki Meilina, Inayati, Jatu Kartika Akmala, Nunung Hidayati, Yusni Khafiyanti, Rahhmawati Ning Rum, Rudy Sigit Kurniawan, Ayu Dita Larasati, Indriana Nurhakimah, Diajeng Kenanga Putri Ma’rifat, Eka Nurul Azizah, Novi Faturohmah, Embun Majiid, Novi Rustiana, Nimas Suri Martiana, Yulita Endah Mayaningrum, Putri Hardiyanti, Noravita.

*Diagnostic*s
Endah Supriyati, Utari Saraswati, Ayu Rahayu, Anwar Rovik, Dian Aruni Kumalawati, Kunta Adi Tetuko, Edwin Widyanto Daniwijaya

Data Management
Yeti Meitika, Dwi Satria Wardhana, Ramdhan Hartanto, Sekar Langit Adesya Paramita, Inggrid Ernesia, Adi Artanto.

Yogyakarta City and Provincial Health Office
Yudiria Amelia, Endang Sri Rahayu, Rubangi, Berti Murtiningsih, Veronika Nur Hardiyati

Trial investigators
Adi Utarini, Citra Indriani, Riris Andono Ahmad, Warsito Tantowijoyo, Eggi Arguni, M. Ridwan Ansari, Benjamin Green, Lauren Hodgson, Zoe Cutcher, Edwige Rancès, Peter A. Ryan, Scott L. O'Neill, Suzanne M. Dufault, Stephanie K. Tanamas, Nicholas P. Jewell, Katherine L. Anders, Cameron P. Simmons

Supplementary Methods

Constrained randomisation procedure

Background

Randomisation is a key step in the design of the AWED cluster randomised trial. Importantly, it will provide a sense of "fairness" and transparency in allocation of the intervention. It is also an opportunity to ensure "balance" between intervention and non-intervention arms. Balance means that, overall, the intervention and non-intervention arms have similar dengue risk apart from the presence of *Wolbachia*. Balance aims to minimise the effect of confounding variables, so that any systematic difference in dengue incidence that occurs between *Wolbachia* intervention areas and untreated areas during the trial can clearly be attributed to the effects of *Wolbachia*. Ensuring a well-balanced trial will reduce the need for statistical adjustment during the analysis phase, increasing the "face validity" of the trial. Balance also improves statistical power and efficiency, reducing the risk of Type II error (i.e. failure to detect an effect when the effect is, in fact, present).[1]

Simple randomisation can be relied upon to produce an overall balance between intervention and non-intervention arms when there is a large number of clusters available for randomisation. However simple randomisation can't be relied on to produce balanced allocations where the sample size is small. For example, in our field efficacy trial involving just 24 clusters, it is possible simple random allocation may allocate most clusters with a high dengue risk to the same treatment arm through chance alone.

Covariate constrained (also referred to as "restricted") randomisation is the best way to achieve balance when the number of clusters is small.[1-3] It ensures balance and minimises loss of statistical power without the need for large numbers of strata. After deciding on appropriate balancing criteria, the approach identifies all potential allocations that will satisfy these criteria, and then randomly selects an allocation pattern from within this restricted list of possibilities. Covariate-constrained randomisation can be used because in general, when assessing the comparability of treatment and control arms, balance is assessed in terms of overall balance between treatment and control arms, rather than

within each matched pair or within each stratum.[2] This approach to randomisation does not generally affect the approach to analysis.[2]

Constrained randomisation for the AWED trial follows guidelines outlined by Hayes and Moulton, 2009.[2] Balancing variables include those that may be potentially confounding covariates; may impact sample size; or may be useful for logistical considerations. Note that balance is necessary at both the cluster and individual levels.[1] Sample size is an important balancing covariate as precision and power is maximised when sample sizes in treatment arms are similar. This includes both the number of clusters and the total number of sampled individuals within each treatment arm.[1]

In Yogyakarta, randomisation was conducted after community-level consent to participate in the RCT was obtained from local leaders (heads of urban villages). This minimises the likelihood that a cluster or part of a cluster declines to allow *Wolbachia* releases after randomisation has occurred and the intervention clusters are known, which would introduce a risk of the study arms becoming unbalanced not only in size but also in other important covariates. *Wolbachia* releases were successfully conducted in all clusters randomised to receive the intervention.

Constraining variables

**Category**	**Constraining variable**	**Data source**	**Balancing criteria**
Potential confounding covariates	Incidence of notified DHF, per 1000 population (2013-15)	Yogyakarta District Health Office	Incidence in each arm deviates from overall incidence by +/- <5%
	Incidence of presentations for other (non-dengue) febrile illness, per 1000 population (2014-15)	18 primary care clinics in Yogyakarta (Puskesmas)	Incidence in each arm deviates from overall incidence by +/- <5%
	% of population aged <15 years	Yogyakarta demographic bureau	Proportion aged <15y in each arm deviates from the overall proportion by +/- <5%
	% of population >20 years that finished school	Yogyakarta demographic bureau	Proportion aged >20y that have finished school in each arm deviates from the overall proportion by +/- <5%

Population at risk	Population (2015)	Yogyakarta Statistics Office (BPS)	Population in each study arm is within 45-55% of the study area total
	Number of clusters per treatment arm	N/A	Fixed at 12 clusters per arm
Logistical considerations	Cluster area (km2)	WMP Yogyakarta	Total area in each study arm is within 45-55% of the study area total
	Cluster non-release area (km2)*	WMP Yogyakarta	Non-release area in each study arm is within 45-55% of the study area total
	Spatial strata	N/A	Each of 4 quadrants of the city will include 6 clusters, 3 intervention and 3 untreated

* to ensure the intervention arm cannot end up with an excess of "non-release" areas

Constrained randomisation method:

1. Calculated values for each balancing covariate for each of the 24 clusters and across the study area as a whole. Cluster values are summarised in Table S1.

2. Generated a large number of potential random allocations (n=100,000)

3. For each allocation, calculated the value of each balancing criterion in each study arm

4. Rejected any random allocations where any one or more of the balancing criteria described in Table 1 were not met.

5. Note that for the potential confounding covariates the comparisons between study arm values and overall values were calculated in two ways, and both were applied as constraints as described above in 4:

    a. Individual-level: the aggregate rate or proportion calculated across the study arm was compared with the aggregate rate or proportion calculated across the whole population

b. Group-level: the mean of 12 cluster-level rates or proportions in the study arm was compared with the mean of all 24 cluster-level rates or proportions.

6. Assessed validity of the scheme:

a. Reviewed restricted number of potential allocations, ensuring the number was not too small relative to the overall number of possible allocations (as above). A minimum threshold of least 100 potential allocations was required.

b. Ensured that, within each stratum, no clusters are NEVER or ALWAYS allocated together, as this would result in an invalid randomisation scheme.

c. Examined dengue incidence correlation over time within clusters frequently randomised together, and compared against correlation within all pairwise combinations. Verified that dengue incidence in clusters frequently randomised together was not highly correlated

7. A total of 244 allocations met the balancing criteria (thus 488 possible distinct randomisations of intervention allocation). A random subset of 100 balanced allocations was selected, as the sampling frame for the final public randomisation event.

8. From this list a single allocation pattern was randomly selected, using numbered balls, at a public participatory event of community and government leaders in Yogyakarta in January 2017.

*w*Mel-infected *Aedes aegypti* mosquito production

An existing colony of local *Ae. aegypti* containing the *w*Mel *Wolbachia* strain, created for the 2016-2017 quasi-experimental study releases in Yogyakarta City,[5] was used as the founder colony for the releases described here. It was backcrossed for three generations with wild-type males collected from the study intervention area to generate the release colony, which was then maintained as described previously.[5] The insecticide resistance profile of the *w*Mel-infected *Ae. aegypti* release material was matched to the local wild-type population as described previously.[5]

Vector competence experiments using *w*Mel-infected *Ae. aegypti* from Yogyakarta

We membrane blood-fed Yogyakarta wild-type (WT) and *w*Mel-infected *Ae. aegypti* at the Oxford University Clinical Research Unit (Vietnam) using viremic blood collected from nine acute dengue inpatients at the Hospital for Tropical Diseases, Ho Chi Minh City, Vietnam using previously published protocols. [6] We measured virological differences between each mosquito line, in abdomen and saliva, at predefined times (10-21 days) after blood feeding. The work was performed under human research ethics approvals from the Hospital for Tropical Diseases, H (HTD CS/ND/12/09 and HTD CS/ND/12/16) and University of Oxford (26DX: OxTREC 68-11 and 30DX: OxTREC 30-12). The virological outcomes (infected/uninfected) are shown in Figure S1. Logistic regression was used to model the probability of successful human-mosquito transmission of DENV in the abdomen and saliva, with mosquito strain (*w*Mel vs wild-type) as the main covariate. Additional analysis adjusted for serotype, donor log viremia, and day of mosquito harvesting. Robust standard errors were used to account for simultaneous exposure of donors to multiple *w*Mel and wild-type *Ae. aegypti*. The difference in DENV copy number between the abdomens of *w*Mel-infected and wild-type *Ae. aegypti* was determined using the Kruskal-Wallis test with Dunn's multiple comparison correction.

After viremic blood-feeding, *w*Mel-infected mosquitoes with this Indonesian genetic background had a slightly reduced probability of having infected abdomens' compared to wild-type *Ae.* aegypti: 57% wMel-infected mosquitoes with abdominal infection vs 70% wild-

type [crude OR=0.57, 95% CI 0.26, 1.26; adjusted for serotype, viremia and day mosquitoes were harvested: OR = 0.27, 95% CI 0.08, 0.92]. Among those mosquitoes with abdomen infections, the DENV viral load in *w*Mel-infected mosquitoes was significantly lower than in wild-type *Ae. aegypti* for each of the three serotypes tested (Figure S2).

As expected, *w*Mel-infected mosquitoes were significantly less likely to have saliva containing infectious virus than their wild-type counterparts: 8% wMel-infected mosquitoes with saliva infection vs 38% wild-type [crude OR=0.14, 95% CI 0.06, 0.34; adjusted for serotype, viremia and day mosquitoes were harvested: OR = 0.07, 95% CI 0.02, 0.33] (Figure S1).

These results are consistent with a large body of work, on a variety of different genetic backgrounds, demonstrating that *w*Mel-infected *Ae. aegypti* have reduced transmission potential for DENV.

*w*Mel-infected *Aedes aegypti* deployments

Wolbachia-carrying mosquitoes were released as eggs using mosquito release containers (MRCs). These were 2-litre plastic buckets each containing one oviposition strip with 100–150 eggs, Tetra Pleco Wafers fish food (Tetra GmbH, Germany), and 1 litre of water. MRCs were covered and placed outside houses, protected from direct sun and rain. Holes drilled near the top of the bucket walls allowed adult mosquitoes to escape. Releases occurred between March and December 2017, with 9–14 rounds of releases in each intervention cluster. Releases stopped in each cluster when the prevalence of *Wolbachia* in field-caught mosquitoes was >60% for three consecutive weeks releases. MRCs were reset every two weeks. An MRC was placed in 1–2 randomly selected locations within each 50x50 m grid square across the intervention area. Permission was obtained from property owners to place MRCs on private property.

Entomological monitoring

Prevalence of *Wolbachia* in the local *Ae. aegypti* population was monitored by weekly collection of adult mosquitoes via a network of 348 BG Sentinel traps (Biogents, Germany).

Written consent was obtained from heads of households hosting BG traps. The median (range) trap density was 16.0 (13.2–18.1) BG/km^2 in the intervention clusters and 14.9 (10.3–16.8) BG/km^2 in the untreated clusters (Table S1). Mosquitoes were demobilised at -20°C for ≥1 hour, then identified by morphological features. The number of mosquitoes caught in each BG trap was recorded by species, sex, and in total. *Ae. aegypti* were stored at -20°C in 80% ethanol until testing for *w*Mel infection.

Field-caught *Ae. aegypti* were screened for *w*Mel *Wolbachia* by qualitative PCR Taqman assay on a Roche LightCycler 480. The qPCR conditions consisted of a denaturation step at 95°C for 5 minutes followed by 45 cycles of PCR (denaturation at 95 °C for 10 seconds, annealing at 60 °C for 15 seconds, and extension at 72 °C for 1 second with the single acquisition) followed by a cooling down step at 40°C for 10 seconds. Specific primers targeting the gene encoding *Ae aegypti Rps17* and *wMel WD0513* were used as previously described,[7] but with replacement of the Cy5-BHQ3 fluorophore-quencher pair in the *w*Mel probe with the fluorophore-quencher LC640-IowaBlack (Integrated DNA technologies).[8] Testing was at weekly intervals when *Wolbachia* prevalence was <80% and 4-weekly intervals when establishment was achieved (≥80% cluster level prevalence for two consecutive testing weeks).

Arbovirus diagnostic investigations

For nucleic acid testing, viral RNA was extracted from participant's plasma samples using a High Pure Viral Nucleic Acid Kit (Roche) according to the manufacturer's instructions. An internally controlled, multiplex real-time RT-PCR assay was used to detect viral RNA from Zika virus (ZIKV), DENV or Chikungunya virus (CHIKV). The internal control was equine arteritis virus (EAV), a standard amount of which was spiked into plasma samples prior to RNA extraction. The primers and probes used for detection of ZIKV,[9] CHIKV,[10] and DENV[11] were as described previously. The primers and probes for DENV targeted the 3' UTR region of the four DENV serotypes. Primers and probes for detection of EAV were as described previously.[12] PCR was performed on a Roche Lightcycler II thermocycler. PCR conditions available from the authors upon request. PCR was performed in batches of test samples and every PCR plate carried external positive controls (supernatants from cultures of DENV-1-4,

CHIK and ZIKV) and negative controls. The DENV serotype specific PCR was performed using a commercial assay (Dengue Simplexa kit (DiaSorin Molecular) on the Liaison MDX thermocycler according to the manufacturer's instructions. The Bio-rad Platelia Dengue NS1 Ag test (BioRad Cat. No. 72830), a one step sandwich format microplate enzyme immunoassay for the detection of DENV NS1 antigen, was performed according to the manufacturer's instructions. The Panbio Dengue IgM Capture Elisa (Abbot Cat. No. 01PE20) and the Panbio Dengue IgG Capture Elisa (Abbot Cat. No. 01PE10) were used to detect IgM and IgG antibodies to dengue antigen in plasma and were used according to the manufacturer's instructions.

Sample size calculations

There are no published formulae to estimate sample size for the proposed study design, ie. a cluster randomised trial with a test-negative design (TND), where the intervention effect is estimated from outcome-based sampling of test-positive and test-negative patients and ascertainment of their exposure status. Randomisation provides a basis of inference in comparing intervention clusters with control clusters as, under the null hypothesis, there should be no difference with regard to the relative appearance of test-positives and negatives in clusters, on average, across the two arms. Thus we proposed as the primary analytical approach a comparison of the exposure odds among test-positive cases versus test-negative controls (for data aggregated across all clusters), with the null hypothesis that the odds of residence in a *Wolbachia*-treated cluster is the same among test-positive cases as test-negative controls. The resulting odds ratio thus provides an estimation of the intervention effect and, as demonstrated previously, provides an unbiased estimate of the relative risk providing that the key assumptions underlying the TND are upheld.

A secondary approach employs as a summary measure for a group-level analysis the proportion of test-positive cases amongst all tested participants in each cluster, with a comparison of the average of these proportions in the intervention arm versus the untreated arm forming the basis of hypothesis testing for intervention effect. The null hypothesis is that the average proportion of total enrolled participants that are cases is the same in treated and untreated study arms. The alternative hypothesis is that the proportion

of enrolled participants that are cases is lower in the *Wolbachia* treated arm than the untreated arm.

Sample size estimations prior to trial commencement

Simulations were used to estimate the power to detect a range of intervention effect sizes using the two methods above, assuming 12 clusters per arm, a fixed total of 1000 true dengue cases enrolled and 4000 non-dengue controls. Empirical data on population, historical dengue incidence and incidence of other febrile illness in the 24 study clusters were used to define the baseline characteristics for the simulated scenarios. Nine overlapping two-year windows of dengue data (2003-2014) were sourced from the Yogyakarta surveillance system. Data for other febrile illness during 2014-2015 were sourced from individual Puskesmas using ICD10 codes for non-localising fever (fever of unknown origin R50; Typhus A75.9; and acute infection due to bacteria at an unspecified site A49). We randomly allocated half the clusters to receive the intervention; this random allocation was repeated one million times, and only those allocations were kept in which the balancing criteria specified in the constrained randomization methods were met (n=244 balanced allocations, and thus 488 possible distinct randomizations of intervention allocation). Dengue case numbers per cluster were either kept at baseline values (for the simulation at the null; ie RR=1) or reduced proportionately (for simulations of intervention effects of RR=0.6, 0.5, 0.4, 0.3). For each of these five 'true' effect sizes, applied to each of the 244 balanced allocations, the 'observed' effect size was calculated from the simulated data by the two methods outlined above; i) aggregated odds ratio for residence in a treated cluster among cases versus controls, and ii) t-test for comparison of the average cluster summary proportions (cases/cases+controls) between study arms. Statistical inference, from the t-test directly, or, for the odds ratios using permutation distribution approximations with standard errors adjusted to account appropriately for the clustered nature of the data, respectively, was used to calculate the proportion of constrained random allocations that yielded a significant result. This provided an estimate of Type I error at the null, and power away from the null (Table). Both of these approaches thus are using approximations to the exact permutation distribution.[4] In practice, the appropriate reference distribution for inference will be based on the set of 244 potential balanced allocations.

These simulations estimated that approximately 1000 cases plus four times as many controls will be sufficient to detect a 50% reduction in dengue incidence with 80% power.

Percent of random allocations that yield significant results on simulated data

Risk Ratio	*T-test*		*Odds Ratio test*	
	Constrained	*Random*	*Constrained*	*Random*
1	0.13	5	1	7
0.6	48	49	61	57
0.5	81	75	89	82
0.4	97	93	99	96
0.3	100	95	100	100

The results show that constrained randomization is somewhat conservative at the null but generally increases power moderately. The odds ratio test is more powerful than the t-test approach, and will thus be used as the primary analysis with the additional attraction of being standard for the traditional test-negative design.

Sample size re-estimation after one year of participant recruitment

A re-estimation of sample size requirements was conducted in January 2019 after one year of recruitment. The initial power calculation used 1000 dengue cases and 4000 non-dengue controls allocated to each cluster based on historical proportions of dengue cases and other febrile illnesses, assuming no variation in the proportion of cases by cluster. This method was found to overestimate power for small samples by not taking into account randomness in the sampling. The sample size re-estimation included power estimates for 200, 400, 600, 800 and 1000 dengue cases with 4 times as many controls. Cases and controls were allocated among clusters by sampling from multinomial distributions, which incorporated

added randomness by allowing the proportion allocated to each cluster to vary across simulations. The re-estimation found that 400 dengue cases plus four times as many controls would be sufficient to detect a 50% reduction in dengue incidence with 80% power.

Power re-estimation for* Wolbachia *contamination scenarios.

Additional simulations were conducted in September 2019 to assess the potential impact on power if a number of untreated clusters were 'lost' to *Wolbachia* contamination. For the target minimum observed effect size of 50% (RR=0.5) and 400 enrolled dengue cases, contamination of 3 untreated clusters (assuming that contaminated clusters experience the full intervention effect for 1 out of the 3 years of trial recruitment) is expected to result in a ~7% loss of power, and contamination of 6 clusters to result in a ~14% loss of power.

Calculation of *Wolbachia* Exposure Index for per-protocol analysis

Participants were asked about their mobility during the period 3 – 10 days prior to illness onset using a structured interview administered at enrolment. This records the duration of time spent at home, work or school, and other locations visited during daylight hours (5am – 9pm) in the 8-day period. The geographic coordinates of those locations were derived by geo-locating them on a digital map, with the assistance of the respondent. A weighted '*Wolbachia* exposure index' (WEI) was defined for each participant as WEI = $\sum(t_j * w_j)$, where t_j is the participant's time spent at location *j* as a proportion of their total observed time, and w_j is the measured cluster-level *Wolbachia* frequency at location *j* in the calendar month of participant enrolment, resulting in a WEI value on a continuous scale from 0 to 1. Aggregate *Wolbachia* prevalence for each cluster was calculated each month from all *Ae. aegypti* trapped in that cluster. For any calendar month where mosquito collection was not done, the average of the cluster-level *Wolbachia* prevalence in the one previous and one subsequent month was used. For visited locations within the quasi-experimental study area, which comprises seven urban villages located adjacent to the northwestern border of the trial study site,[5] the measured village-level *Wolbachia* prevalence from the screening event closest in time to the participant's enrolment was used. Visited locations outside of both the AWED study area and the quasi-experimental study area were assumed to have a

Wolbachia prevalence of zero. The process of calculating WEI was conducted blinded to participants' case/control status, by partitioning the travel history data from the laboratory diagnostic data, to remove any possibility of observer bias.

An additional per-protocol analysis calculated WEI using only the cluster-level *Wolbachia* prevalence in the participant's cluster of residence (in the calendar month of participant enrolment), ignoring the participant's recent travel history. This recognises that dengue exposure risk may be higher at home versus other locations, rather than assuming an even distribution of exposure risk across daytime hours and locations visited.

Per-protocol analysis statistical methods and results

Cases and controls were classified by strata of their WEI: 0-<0.2; 0.2-<0.4; 0.4-<0.6; 0.6-<0.8; and 0.8-1. This acknowledges that the WEI is not a highly precise measure, and serves to reduce error in exposure classification. The ITT methods described were extended to allow for this individual level covariate using a generalized linear model with the cluster bootstrap based on 1,000 clustered resamples. Balanced bootstrap resampling based on cluster membership accommodates within cluster dependencies and has been shown to be a competitive alternative to GEE and mixed effects approaches in the analysis of hierarchical data.[13] Such models yield an estimate, and associated bootstrap percentile-based confidence interval, for the relative risk. Efficacy was then calculated as 100*(1-RR). The WEI strata was included as an unordered covariate to calculate stratum-specific IRRs (relative to the baseline 0-<0.2 stratum). The model results suggest a threshold effect (rather than a "linear" dose-response). For this reason, the WEI strata was not treated as an ordinal covariate, though this had been discussed in the SAP.

There exist two other modifications from the approach described in the SAP. First, the presented model results do not include adjustment for time. Second, the original analysis plan proposed the use of generalized linear mixed effects models with a random effect for cluster membership to account for hierarchical dependencies. However, the resulting point efficacy estimates were not robust to the proposed cluster random effect. As such, the generalized linear model framework is maintained, but with a clustered bootstrap approach to estimation and inference, thereby avoiding the bias and statistical inefficiency introduced from misspecification of the random effects distribution.

Supplementary Tables

Table S1: Baseline characteristics of clusters (sociodemographic, historical disease incidence, area and mosquito trapping)

	Population (2015), N	Population aged <15 years, %	Completed high school, %	Notified DHF incidence per 10,000 (2013-15)	Other febrile illness incidence per 10,000 (2014-15)	Total cluster area, km^2	Residential (release) area, km^2	BG trap density per km^2 of residential (release) area
Intervention	*153,403*	*22*	*74*	*19.7*	*199.3*	*12.24*	*11.04*	*15.7*
Cluster 1	12,747	21	56	19.3	210.0	0.94	0.88	15.9
Cluster 2	10,179	21	78	19.3	91.2	0.69	0.69	14.5
Cluster 6	22,085	20	78	15.3	175.3	0.94	0.93	15.1
Cluster 7	19,587	21	79	11.9	185.8	1.11	0.98	16.3
Cluster 9	13,132	21	83	17.2	205.1	1.14	1.06	13.2
Cluster 10	8,127	22	60	31.4	268.2	1.17	1.00	16.0
Cluster 12	18,947	20	85	15.8	113.4	1.13	1.08	16.7
Cluster 14	15,101	21	83	18.4	196.4	1.27	1.14	14.9
Cluster 16	10,474	22	79	22.6	333.3	0.89	0.86	16.3
Cluster 19	9,726	22	84	20.9	75.5	0.83	0.83	15.7
Cluster 21	8,348	23	55	27.5	385.0	1.04	0.87	16.1
Cluster 24	4,950	24	61	17.1	153.0	1.09	0.72	18.1
Untreated	*158,279*	*22*	*77*	*19.0*	*215.6*	*13.38*	*12.13*	*14.3*
Cluster 3	17,702	21	78	16.3	372.8	1.02	0.92	16.3
Cluster 4	6,471	20	61	13.2	228.5	0.93	0.73	15.1
Cluster 5	12,936	21	81	22.7	112.5	1.1	1.06	12.3

Cluster 8	16,026	21	76	16.9	241.7	1.12	1.08	14.8
Cluster 11	14,983	21	79	19.6	120.9	0.86	0.8	12.5
Cluster 13	28,541	21	80	13.5	158.0	1.64	1.54	15.6
Cluster 15	8,976	22	82	17.9	182.9	0.95	0.87	10.3
Cluster 17	4,031	23	90	19.5	390.9	0.73	0.63	15.9
Cluster 18	21,185	21	76	17.3	113.3	1.6	1.43	16.8
Cluster 20	10,780	22	91	28.0	100.7	1.03	1.01	15.8
Cluster 22	9,971	23	69	23.9	416.3	1.33	1.16	12.9
Cluster 23	6,677	24	63	19.0	148.5	1.07	0.9	13.3

Table S2: Summary of *Wolbachia* deployments in 12 intervention clusters

Cluster	Total area (km^2)	Release start and end date	# release rounds	Mean release points per round (min, max)	Mean estimated mosquitoes released per round (st dev)
1	0.94	6 Mar – 18 Sept 2017	12	342 (267, 382)	22,180 (2959)
2	0.69	13 Mar – 10 Oct 2017	13	280 (268, 313)	18,192 (3512)
6	0.94	17 Apr – 16 Nov 2017	13	426 (382, 518)	28,192 (3996)
7	1.11	17 May – 14 Nov 2017	11	509 (456, 653)	31,319 (4595)
9	1.14	1 May – 13 Nov 2017	12	453 (414, 542)	30,217 (4311)
10	1.17	15 Apr – 26 Sep 2017	9	408 (390, 449)	27,825 (3953)
12	1.13	11 May – 6 Dec 2017	13	484 (441, 586)	33,966 (4557)
14	1.27	10 Apr – 21 Nov 2017	14	515 (478, 616)	34,055 (2519)
16	0.89	29 Mar – 7 Nov 2017	14	408 (374, 505)	26,004 (3751)
19	0.83	20 Mar – 17 Oct 2017	13	349 (326, 393)	22,971 (2968)
21	1.04	6 Apr – 3 Oct 2017	11	409 (400, 425)	26,705 (2577)
24	1.09	25 May – 5 Dec 2017	12	365 (323, 447)	23,328 (3066)

Table S3: Baseline characteristics of trial participants

	N	Age, median (IQR)	Female sex, n (%)
Total enrolled participants	8144	11.6 (6.7, 20.9)	3973 (48.8)
**By inclusion in analysis dataset**			
Included in analysis	6306	12.0 (7.0, 21.1)	3073 (48.7)
Excluded from analysis	1838	10.5 (6.1, 19.4)	900 (49.0)
**By treatment allocation***			
Intervention arm	2905	12.0 (6.9, 21.1)	1430 (49.2)
Untreated arm	3401	12.0 (7.0, 21.2)	1643 (48.3)
**By diagnostic outcome****			
VCD cases	385	11.0 (7.2, 18.1)	189 (49.1)
VCC cases	4	28.5 (16.0, 42.3)	1 (25.0)
Test-negative controls	5921	12.1 (7.0, 21.4)	2884 (48.7)
**By follow-up status***			
Not hospitalized	5567	12.1 (7.1, 21.2)	2707 (48.6)
Hospitalised	295	11.9 (6.9, 20.5)	138 (46.8)
Died		-	-
Lost to follow-up	444	10.8 (6.4, 20.8)	22 (51.4)

Age is in years. IQR: interquartile range, VCD: virologically-confirmed dengue, VCC: virologically-confirmed chikungunya. *Participants in analysis dataset only. **Participants in analysis dataset plus 4 chikungunya cases.

Table S4: Hospitalisation outcome by treatment arm for all participants in the analysis dataset

	Intervention arm		Untreated arm		OR (95% CI)
	N	%	N	%	
**All participants**					
Not hospitalized	2602	89.6	2965	87.2	Ref
Hospitalised	81	2.8	214	6.3	0.43 (0.32, 0.58)
Lost to follow-up	222	7.6	222	6.5	-

Table S5: Hospitalisation outcome by treatment arm for VCD cases

	Intervention arm		Untreated arm	
	N	%	N	%
**VCD**				
Not hospitalised	52	77.6	197	61.9
Hospitalised	13	19.4	102	32.1
Lost to follow-up	2	3.0	19	6.0

Supplementary Figures

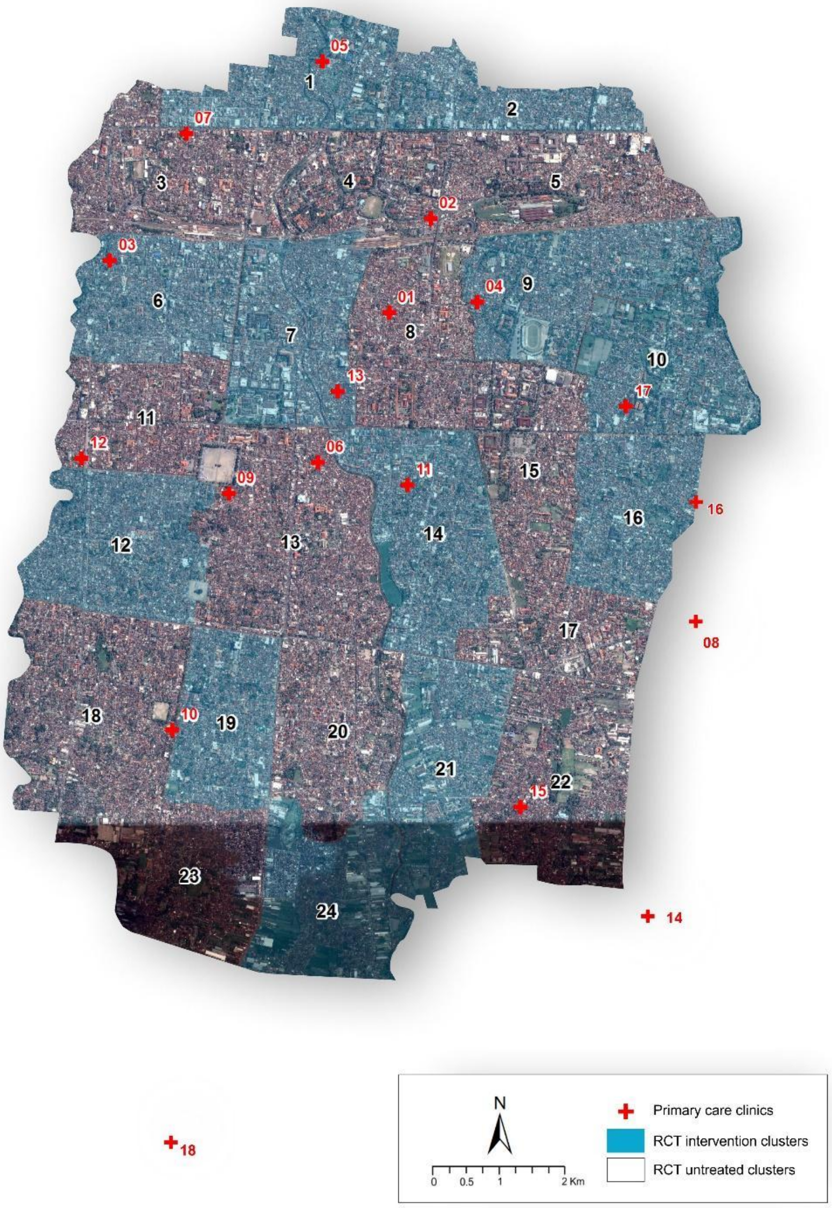

Figure S1: Map of study location with satellite overlay.

The map of Yogyakarta City (plus a small region of neighbouring Bantul District) is shown with wMel intervention clusters (shaded blue) and untreated clusters (no shading) indicated. The locations of primary care clinics (red crosses) where enrolment occurred are also shown, and are numbered to correspond with the clinical-level data in Figure S5.

			Abdomen								Saliva							
			Day 10		Day 14		Day 18		Day 21		Day 10		Day 14		Day 18		Day 21	
Serotype	Donor log viremia	Donor ID	*w*Mel	WT	*w*Mel	WT	*w*Mel	WT	*w*Mel	WT	*w*Mel	WT	*w*Mel	WT	*w*Mel	WT	*w*Mel	WT
DENV-1	7.63	43	5/8	7/8	2/10	10/10	4/8	8/8	7/7	7/7	1/8	4/8	0/10	10/10	0/8	8/8	4/7	7/7
	8.32	35	8/8	8/8	10/10	5/5	22/22	7/7			1/8	2/8	1/10	1/5	5/22	4/7		
DENV-2	8.57	50	8/8	8/8	9/11	10/11					0/8	1/8	2/11	6/11				
	9.05	41	7/8	7/8	9/10	10/10	8/8	8/8			0/8	2/8	0/10	7/10	0/8	7/8		
DENV-3	6.69	52	0/8	0/8	0/10	0/10	0/8	0/8	0/2	1/2	0/8	0/8	0/10	0/10	0/8	0/8	0/2	0/1
DENV-4	7.10	47	0/8	0/8	0/10	0/10	0/3	0/3			0/8	0/8	0/10	0/10	0/3	0/3		
	7.65	34	8/8	7/8	10/10	10/10	13/13	14/14			0/8	4/8	2/10	6/10	3/13	5/14		
	7.93	39	0/8	5/8	0/10	4/10					0/8	2/8	0/10	1/10				
	8.81	44	2/8	7/8	0/6	5/6					0/8	0/8	0/6	4/6				

Figure S2. Susceptibility of wild-type [WT] and wMel-infected Ae. aegypti to DENV infection. Each row represents the results of feeding cohorts of WT and *w*Mel-infected mosquitoes on viremic blood collected from human dengue cases. The infecting DENV serotype and $\log_{10}$ viral titre (RNA copies/ml) of the donor blood is shown (also indicated by the horizontal blue bars). Results indicate the numbers of mosquitoes with detectable DENV infection in the abdomen and saliva over the total number of mosquitoes fed on blood from that donor and harvested at four time points after feeding (days 10, 14, 18 and 21). Background colour of table cells indicates the proportion of mosquitoes with detectable infection (0%: dark green to 100%: dark red).

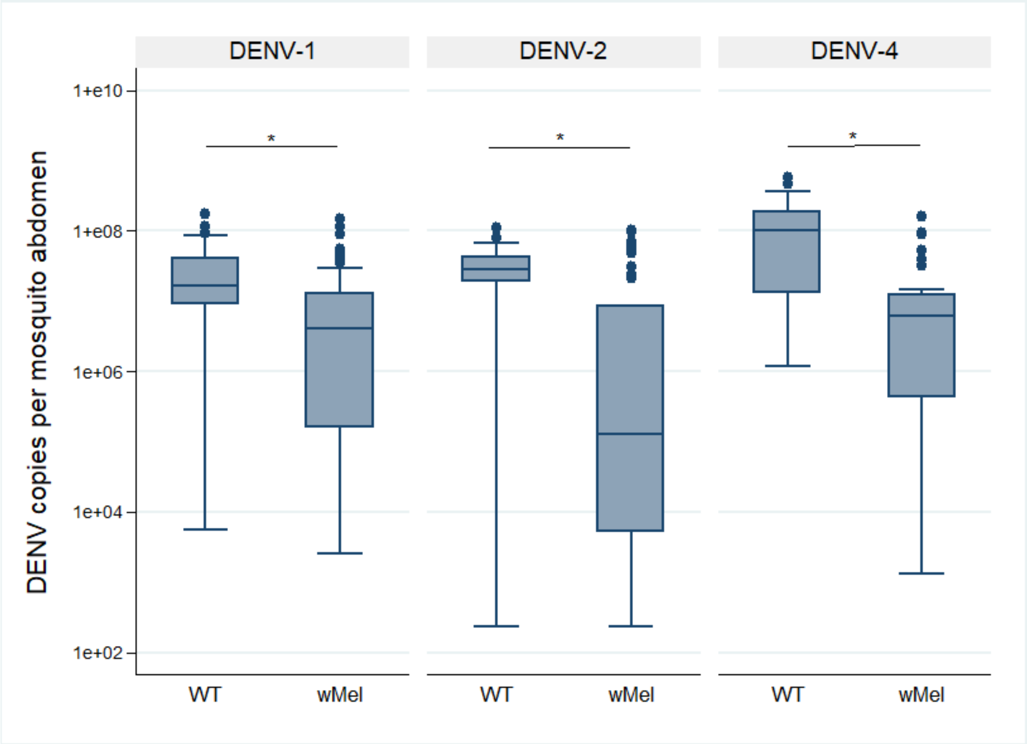

Figure S3: Viral load in mosquito abdomen tissue per mosquito by strain (*w*Mel vs wild-type [WT]) and serotype.

DENV-3 was excluded as only one mosquito tested positive for DENV in the abdomen. Boxes are median and interquartile range for DENV copies per mosquito. Whiskers indicate the range, and circles indicate outliers. *p<0.001 for Kruskal-Wallis test for difference in viral copy number, with Dunn's multiple comparison correction.

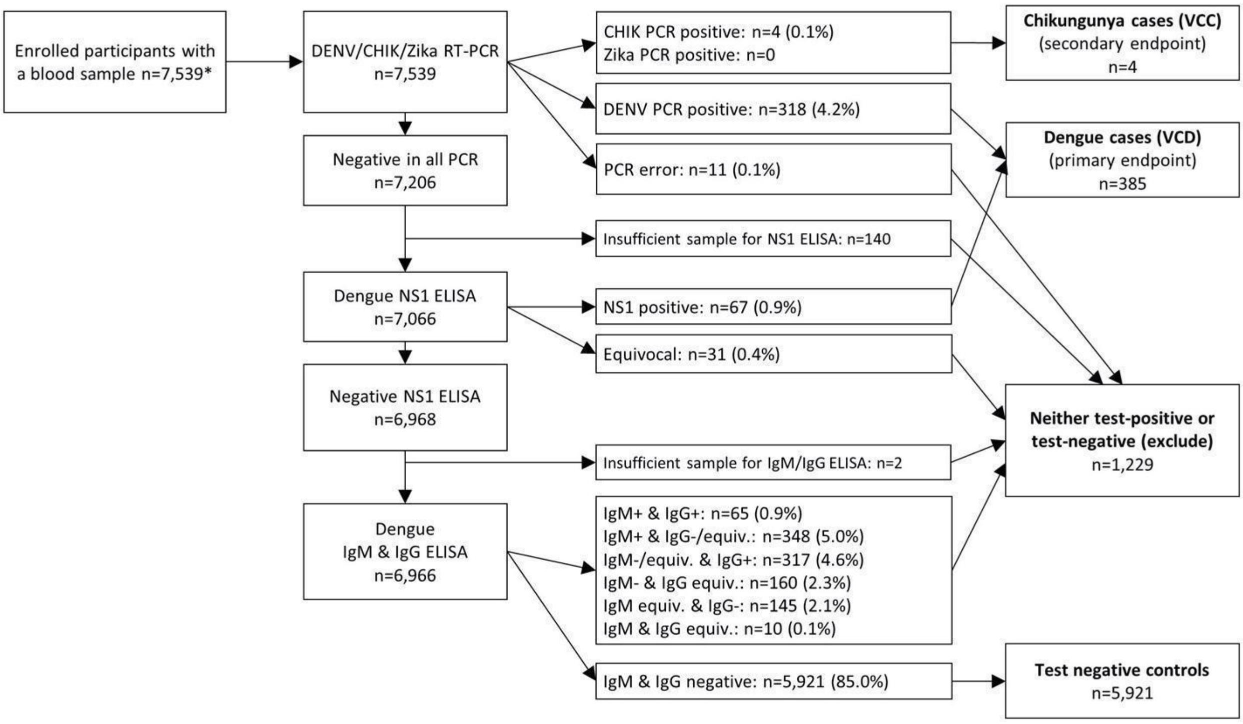

*Enrolled after *Wolbachia* establishment (i.e. 8 Jan 2018); excludes 186 test-negative controls enrolled in a month with no test-positive dengue cases (September 2018)

Figure S4: Diagnostic algorithm and classification of participants

Among 8,144 total participants enrolled, 7,539 were enrolled after *Wolbachia* establishment (8 Jan 2018) and with a blood sample successfully collected, excluding 186 test-negative controls (95 in the intervention arm and 91 in the untreated arm) who were enrolled in a month with no test-positive dengue cases (September 2018). Four virologically-confirmed chikungunya (VCC) and 318 virologically-confirmed dengue (VCD) cases were identified by multiplex RT-PCR. Another 67 VCD cases were negative in RT-PCR but positive in dengue NS1 antigen enzyme-linked ELISA. 1,229 participants were classified as neither test-positive cases nor test-negative controls due to a positive or equivocal result for dengue IgM and/or IgG antibody, or due to insufficient sample volume for complete diagnostics or an inconclusive diagnostic result. 5921 participants with negative results in all diagnostic tests were classified as test-negative controls.

DENV: dengue virus; CHIK: chikungunya virus; Zika: Zika virus; PCR: polymerase chain reaction; VCC: virologically-confirmed chikungunya; VCD: virologically-confirmed dengue; NS1: dengue non-structural protein 1; ELISA: enzyme-linked immunosorbent assay; IgM: immunoglobulin M; IgG: immunoglobulin G.

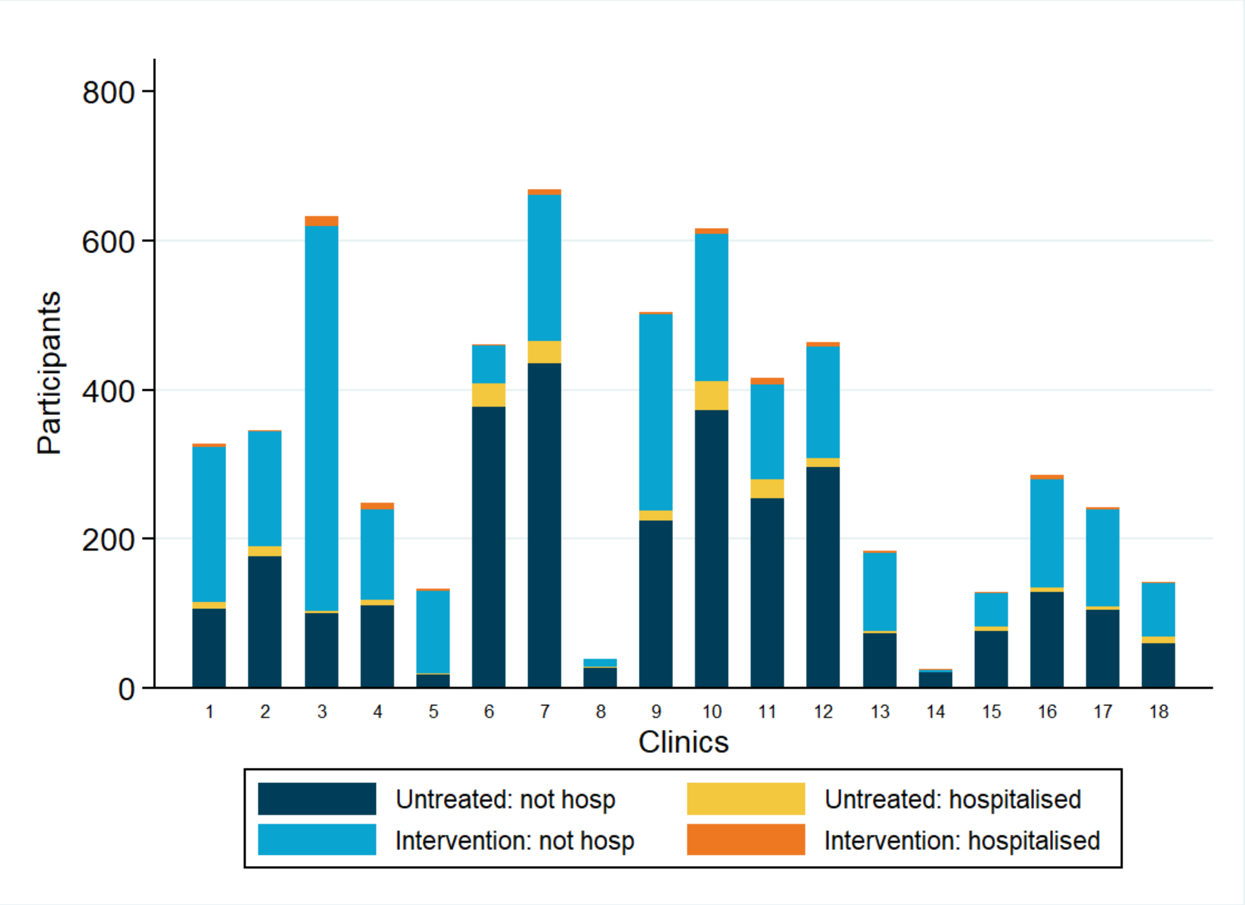

Figure S5: Participant enrolment and hospitalisations by primary care clinic and treatment arm.

Bars show the number of participants in the analysis dataset by clinic of enrolment (clinics numbered 1 - 18). Each clinic enrolled participants from both untreated (dark blue; yellow) and *Wolbachia*-treated (light blue; orange) clusters. The proportion of participants who were hospitalised varied between clinics, and was higher among participants from untreated (yellow) than *Wolbachia*-treated (orange) clusters.

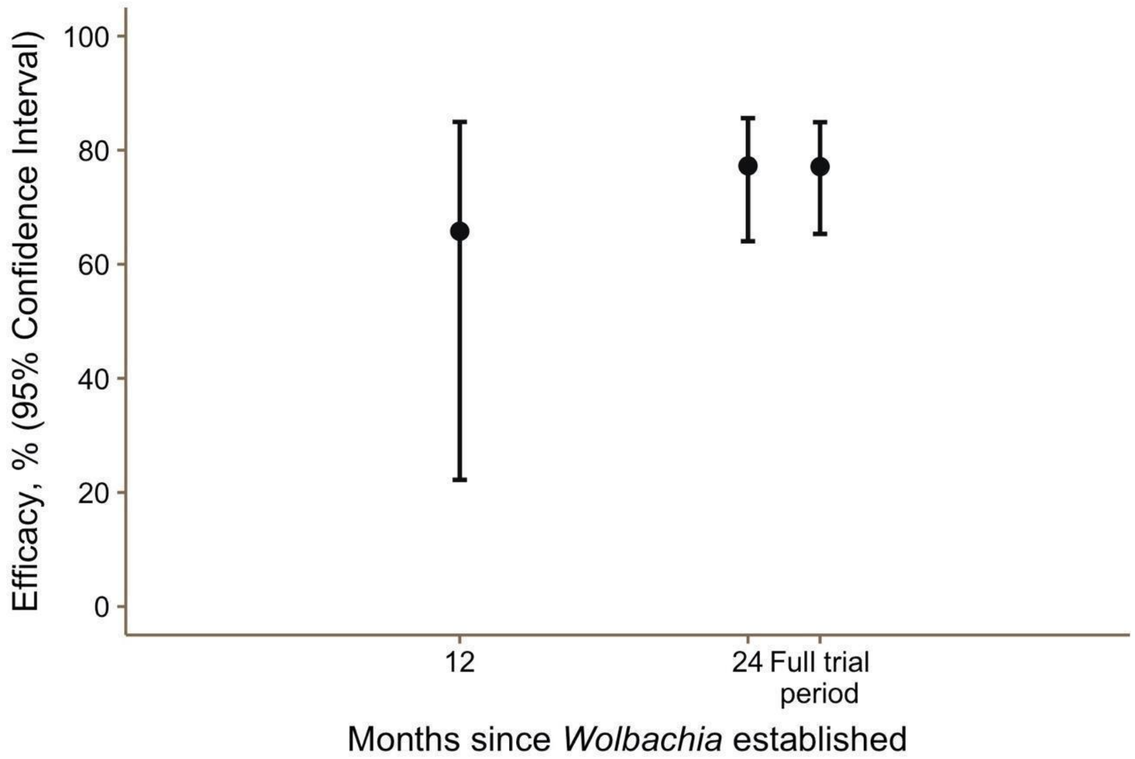

Figure S6: Intervention efficacy over time.

Efficacy is calculated as 100*(1-aggregate odds ratio) among participants enrolled within the first 12 months after *w*Mel establishment, within the first 24 months, and within the full 27 month trial period.

A

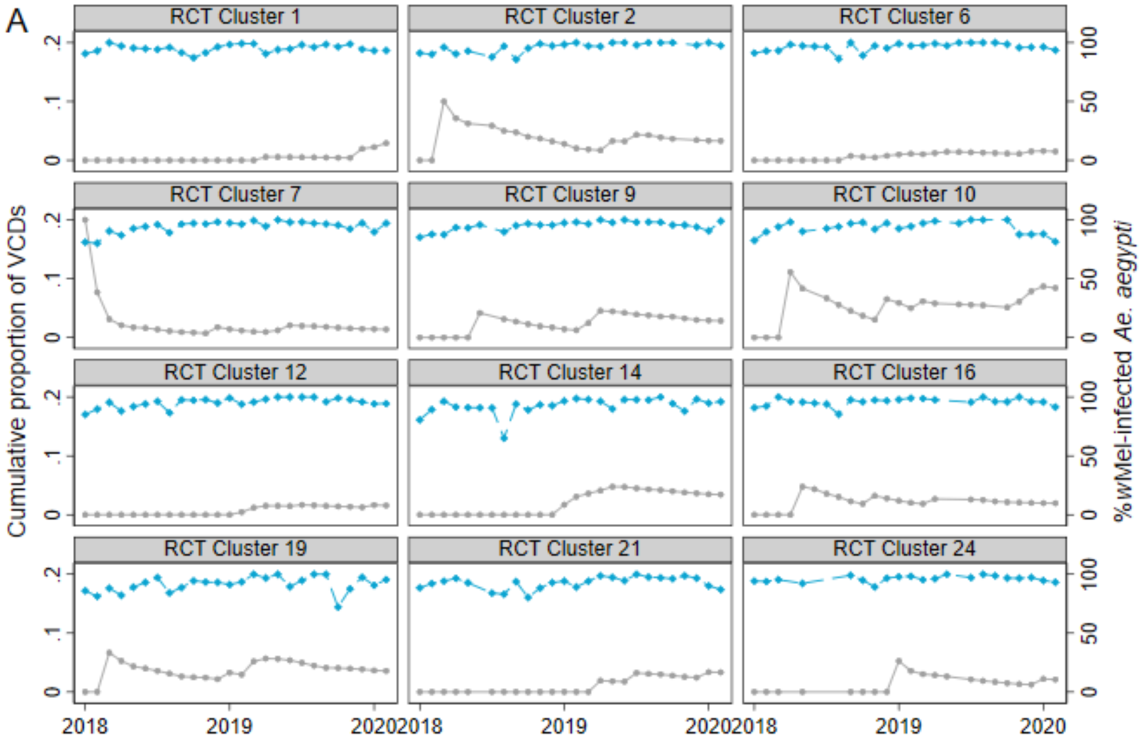

RCT Cluster 1
RCT Cluster 2
RCT Cluster 6
RCT Cluster 7
RCT Cluster 9
RCT Cluster 10
RCT Cluster 12
RCT Cluster 14
RCT Cluster 16
RCT Cluster 19
RCT Cluster 21
RCT Cluster 24
Cumulative proportion of VCDs
%wMel-infected Ae. aegypti
0
.1
.2
0
50
100
2018
2019
2020

B

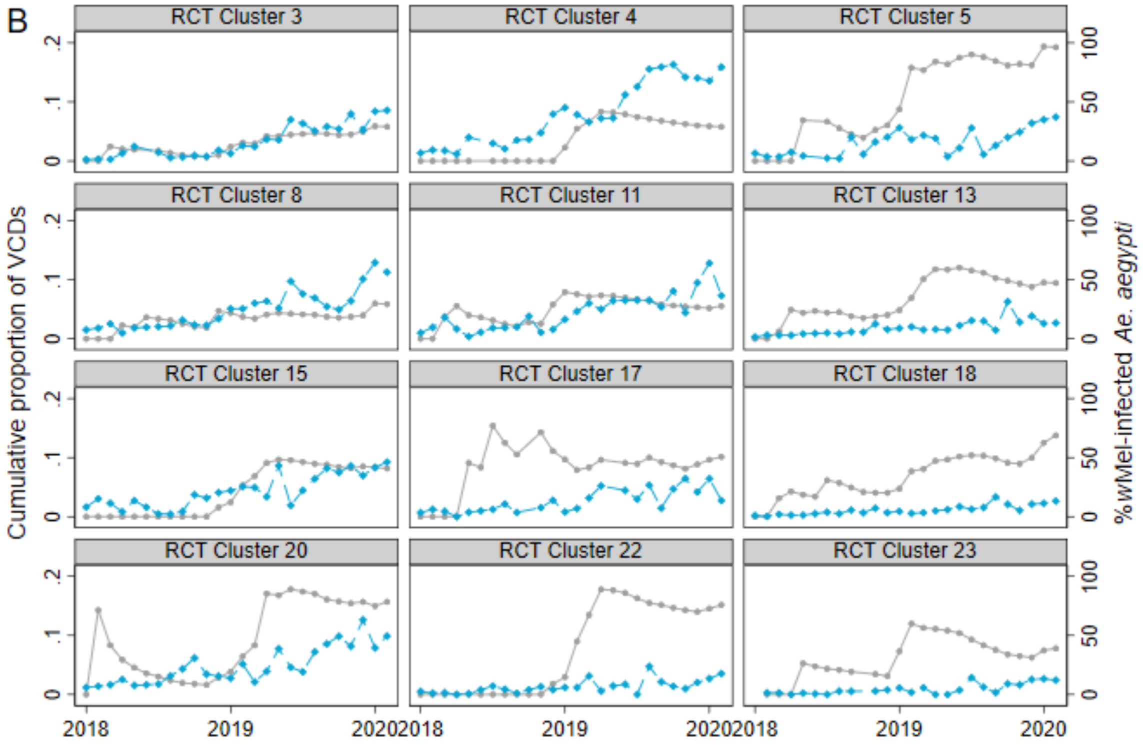

RCT Cluster 3
RCT Cluster 4
RCT Cluster 5
RCT Cluster 8
RCT Cluster 11
RCT Cluster 13
RCT Cluster 15
RCT Cluster 17
RCT Cluster 18
RCT Cluster 20
RCT Cluster 22
RCT Cluster 23
Cumulative proportion of VCDs
%wMel-infected Ae. aegypti
0
.1
.2
0
50
100
2018
2019
2020

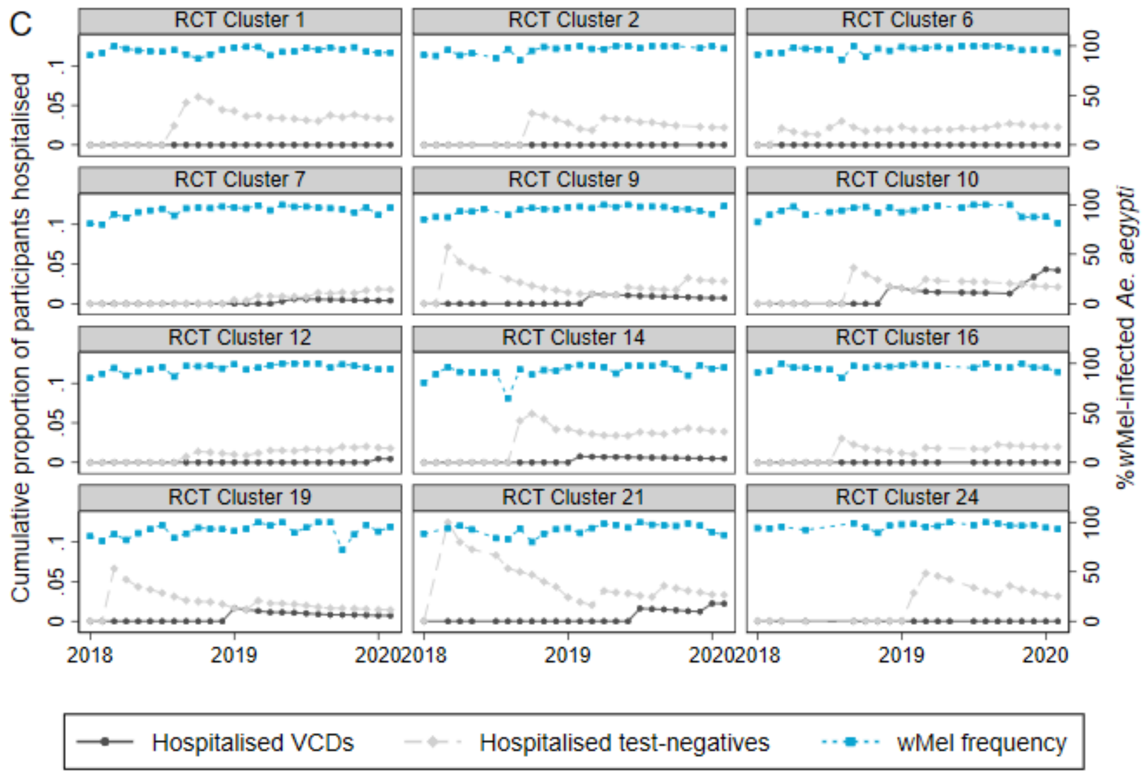
C
RCT Cluster 1
RCT Cluster 2
RCT Cluster 6
RCT Cluster 7
RCT Cluster 9
RCT Cluster 10
RCT Cluster 12
RCT Cluster 14
RCT Cluster 16
RCT Cluster 19
RCT Cluster 21
RCT Cluster 24
Cumulative proportion of participants hospitalised
%wMel-infected Ae. aegypti
2018
2019
2020
Hospitalised VCDs
Hospitalised test-negatives
wMel frequency

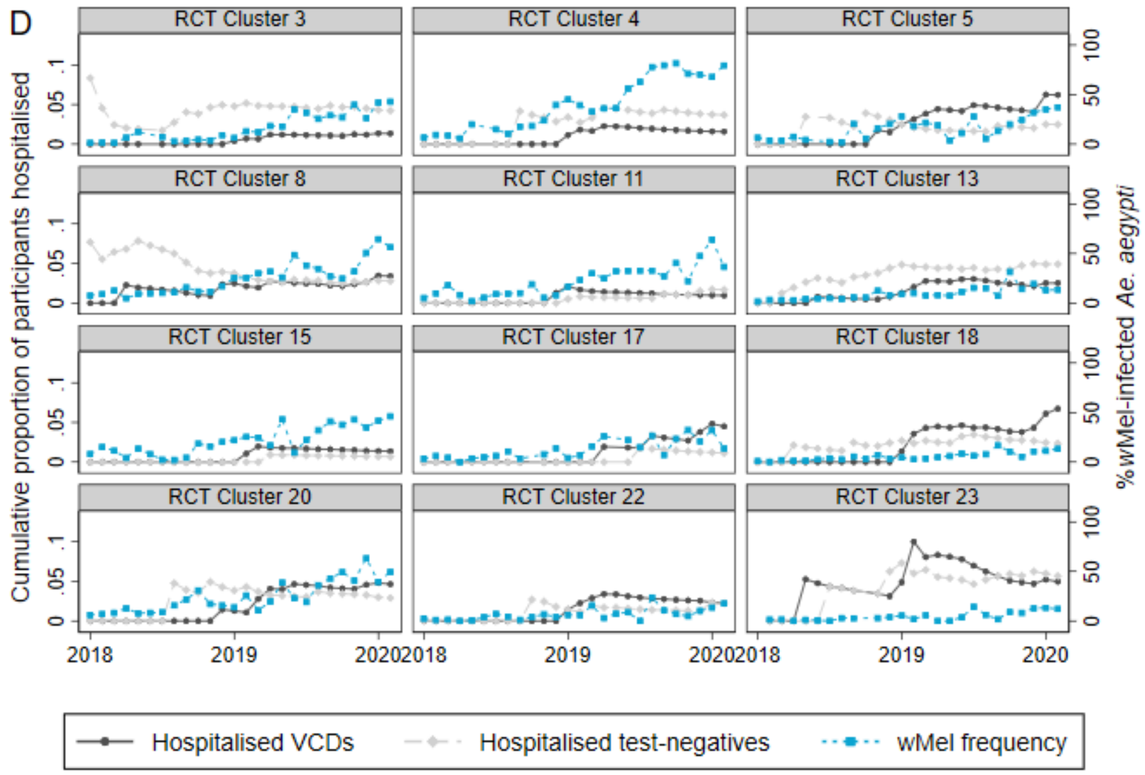
D
RCT Cluster 3
RCT Cluster 4
RCT Cluster 5
RCT Cluster 8
RCT Cluster 11
RCT Cluster 13
RCT Cluster 15
RCT Cluster 17
RCT Cluster 18
RCT Cluster 20
RCT Cluster 22
RCT Cluster 23
Cumulative proportion of participants hospitalised
%wMel-infected Ae. aegypti
2018
2019
2020
Hospitalised VCDs
Hospitalised test-negatives
wMel frequency

Figure S7: Cluster-level proportions of virologically-confirmed dengue cases (A and B), proportions of participants hospitalised (C and D), and prevalence of *w*Mel in local *Aedes aegypti* mosquito populations over time. For each of 12 intervention clusters (A and C) and 12 untreated clusters (B and D), the grey markers show VCD cases as a proportion of all participants or participants hospitalised as a proportion of all participants (cumulative to each month January 2018 - March 2020). Panel C excludes one observation from cluster 21 (February 2018) where the proportion of hospitalised test-negative participants was 0.2. The blue markers show the percentage of *Ae. aegypti* collected in each cluster in each month that were *w*Mel infected.

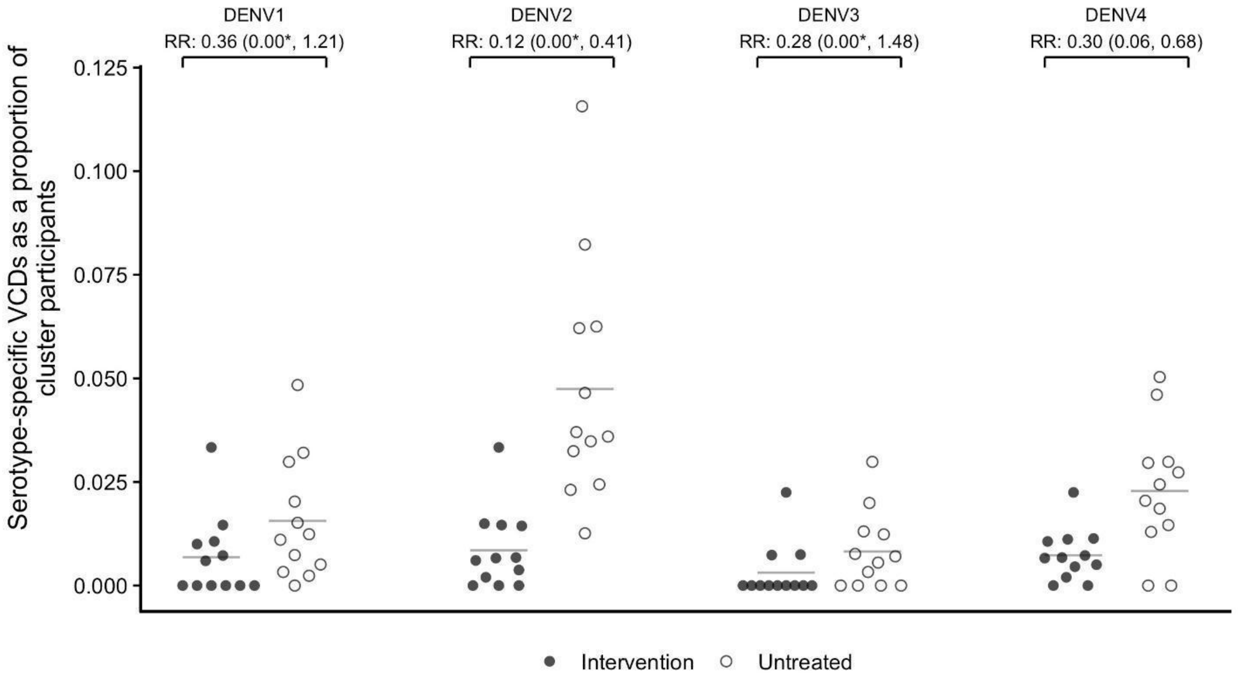

Figure S8: Cluster-level proportions of serotype-specific virologically-confirmed dengue cases.

Serotype-specific VCD cases as a proportion of all participants in *Wolbachia*-treated (closed circles) and untreated (open circles) clusters. Horizontal bars show the mean serotype-specific VCD proportion in intervention and untreated clusters; the relative risk and confidence intervals are derived from a comparison of these mean proportions (see Methods). Confidence intervals have not been adjusted for multiple comparisons. *When the number of serotype-specific VCD cases is small while the number of test-negative controls remains large, the test-positive fraction method is not sufficiently precise to distinguish between very low values of the relative risk that are close to zero; in this case the method yields a lower confidence interval of 0 as reported here.

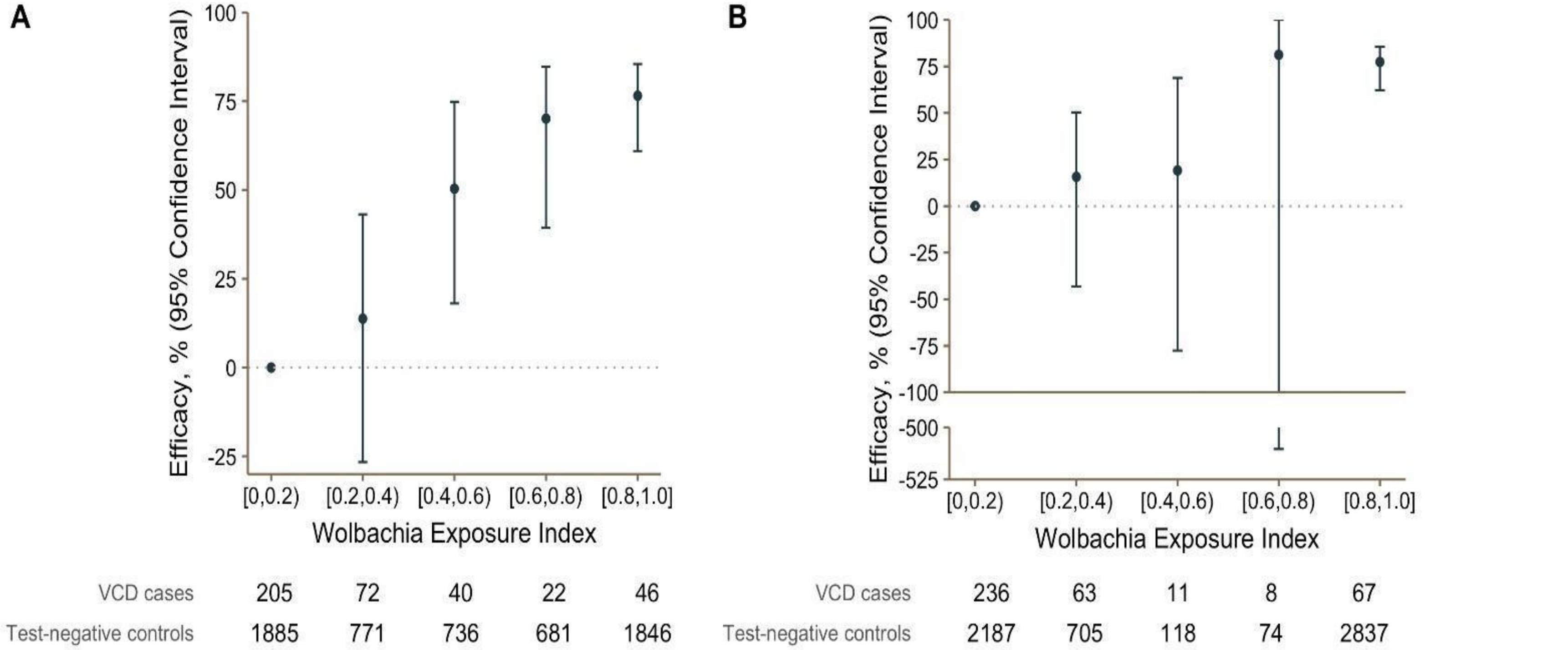

Figure S9: Efficacy of the *Wolbachia* intervention against virologically-confirmed dengue according to *Wolbachia* exposure index (per-protocol analysis).

Markers show stratum-specific efficacy (and 95% confidence intervals) against VCD by quintile of *Wolbachia* exposure index, with WEI based on A) duration-weighted *w*Mel frequencies in the cluster of residence and other visited locations, or B) *w*Mel frequency in cluster of residence only. The number of VCD cases and test-negative controls with WEI values in each quintile is shown beneath the plots.

References

1. Ivers NM, Halperin IJ, Barnsley J, et al. Allocation techniques for balance at baseline in cluster randomized trials: a methodological review. Trials 2012;13:120.

2. Hayes RJ, Moulton LH. Cluster randomized trials. London: Chapman & Hall/CRC Press; 2009.

3. Smith PG, Morrow RH, Ross DA, eds. Field trials of health interventions: a toolbox (3rd ed.): Oxford University Press; 2015.

4. Jewell NP, Dufault S, Cutcher Z, Simmons CP, Anders KL. Analysis of cluster-randomized test-negative designs: cluster-level methods. Biostatistics 2019;20:332-46.

5. Indriani C, Tantowijoyo W, Rances E, et al. Reduced dengue incidence following deployments of *Wolbachia*-infected *Aedes aegypti* in Yogyakarta, Indonesia: a quasi-experimental trial using controlled interrupted time series analysis. Gates Open Res 2020;4:50.

6. Carrington LB, Tran BCN, Le NTH, et al. Field- and clinically derived estimates of Wolbachia-mediated blocking of dengue virus transmission potential in Aedes aegypti mosquitoes. Proc Natl Acad Sci U S A 2018;115:361-6.

7. Yeap HL, Axford JK, Popovici J, et al. Assessing quality of life-shortening *Wolbachia*-infected *Aedes aegypti* mosquitoes in the field based on capture rates and morphometric assessments. Parasit Vectors 2014;7:58.

8. Dar M, Giesler T, Richardson R, et al. Development of a novel ozone- and photo-stable HyPer5 red fluorescent dye for array CGH and microarray gene expression analysis with consistent performance irrespective of environmental conditions. BMC Biotechnol 2008;8:86.

9. Faye O, Faye O, Diallo D, Diallo M, Weidmann M, Sall AA. Quantitative real-time PCR detection of Zika virus and evaluation with field-caught mosquitoes. Virol J 2013;10:311.

10. Pastorino B, Bessaud M, Grandadam M, Murri S, Tolou HJ, Peyrefitte CN. Development of a TaqMan RT-PCR assay without RNA extraction step for the detection and quantification of African Chikungunya viruses. J Virol Methods 2005;124:65-71.

11. Rances E, Ye YH, Woolfit M, McGraw EA, O'Neill SL. The relative importance of innate immune priming in *Wolbachia*-mediated dengue interference. PLoS Pathog 2012;8:e1002548.

12. Hue KD, Tuan TV, Thi HT, et al. Validation of an internally controlled one-step real-time multiplex RT-PCR assay for the detection and quantitation of dengue virus RNA in plasma. J Virol Methods 2011;177:168-73.

13. Deen M, de Rooij M. ClusterBootstrap: An R package for the analysis of hierarchical data using generalized linear models with the cluster bootstrap. Behav Res Methods 2020;52:572-90
